# Immuno-inflammatory *in vitro* hepatotoxicity models to assess side effects of biologicals exemplified by aldesleukin

**DOI:** 10.3389/fimmu.2023.1275368

**Published:** 2023-11-17

**Authors:** Luise A. Roser, Sonja Luckhardt, Nicole Ziegler, Dominique Thomas, Pia Viktoria Wagner, Georg Damm, Andrea Scheffschick, Philip Hewitt, Michael J. Parnham, Susanne Schiffmann

**Affiliations:** ^1^ Department of Preclinical Research, Fraunhofer Institute for Translational Medicine and Pharmacology (ITMP), Frankfurt am Main, Germany; ^2^ pharmazentrum frankfurt/ZAFES, Department of Clinical Pharmacology, Goethe-University Hospital Frankfurt, Frankfurt am Main, Germany; ^3^ Department of Hepatobiliary Surgery and Visceral Transplantation, University Hospital, Leipzig University, Leipzig, Germany; ^4^ Chemical and Preclinical Safety, Merck Healthcare KGaA, Darmstadt, Germany; ^5^ Fraunhofer Cluster of Excellence Immune-Mediated Diseases (CIMD), Frankfurt am Main, Germany

**Keywords:** immune-related adverse events, immunotherapy, *in vitro* prognostics, triple-culture model, immune cells

## Abstract

**Introduction:**

Hepatotoxicity induced by immunotherapeutics is an appearing cause for immune-mediated drug-induced liver injury. Such immuno-toxic mechanisms are difficult to assess using current preclinical models and the incidence is too low to detect in clinical trials. As hepatotoxicity is a frequent reason for post-authorisation drug withdrawal, there is an urgent need for immuno-inflammatory *in vitro* models to assess the hepatotoxic potential of immuno-modulatory drug candidates. We developed several immuno-inflammatory hepatotoxicity test systems based on recombinant human interleukin-2 (aldesleukin).

**Methods:**

Co-culture models of primary human CD8^+^ T cells or NK cells with the hepatocyte cell line HepaRG were established and validated with primary human hepatocytes (PHHs). Subsequently, the HepaRG model was refined by increasing complexity by inclusion of monocyte-derived macrophages (MdMs). The main readouts were cytotoxicity, inflammatory mediator release, surface marker expression and specific hepatocyte functions.

**Results:**

We identified CD8^+^ T cells as possible mediators of aldesleukin-mediated hepatotoxicity, with MdMs being implicated in increased aldesleukin-induced inflammatory effects. In co-cultures of CD8^+^ T cells with MdMs and HepaRG cells, cytotoxicity was induced at intermediate/high aldesleukin concentrations and perforin was upregulated. A pro-inflammatory milieu was created measured by interleukin-6 (IL-6), c-reactive protein (CRP), interferon gamma (IFN-γ), and monocyte chemoattractant protein-1 (MCP-1) increase. NK cells responded to aldesleukin, however, only minor aldesleukin-induced cytotoxic effects were measured in co-cultures. Results obtained with HepaRG cells and with PHHs were comparable, especially regarding cytotoxicity, but high inter-donor variations limited meaningfulness of the PHH model.

**Discussion:**

The *in vitro* test systems developed contribute to the understanding of potential key mechanisms in aldesleukin-mediated hepatotoxicity. In addition, they may aid assessment of immune-mediated hepatotoxicity during the development of novel immunotherapeutics.

## 1 Introduction

Drug-induced liver injury (DILI) is a threat to patient health and a major reason for drug withdrawal after approval (1). Current preclinical testing does not reliably detect drug-induced hepatotoxicity and the incidence of this adverse reaction is too rare to identify it in pre-marketing clinical trials (2). With augmenting numbers of immunomodulatory therapies receiving marketing authorisation for the treatment of immune system disorders and cancer, immune-mediated injury mechanisms play an increasingly important role in DILI (3).

Drugs can damage the liver via multiple mechanisms. Direct (intrinsic) hepatotoxicity is the most common DILI type. It is induced by the inherent toxic effects of the drug, and is dose-dependent and predictable, with acetaminophen-induced injury as prominent example. Idiosyncratic DILI is caused by the uptake, metabolism or transport of the drug, e.g. by the patients’ genetic or metabolic predispositions and their immune system. It is unpredictable and no animal models for idiosyncratic DILI are available. Drug classes linked to idiosyncratic DILI are for instance antimicrobial antibiotics and psychoactive drugs (4, 5). Indirect hepatotoxicity is an emerging subclass of DILI and occurs with a higher frequency than idiosyncratic DILI. It is generated by the effect(s) the drug induces in the body and not by the molecules’ inherent toxic or immunogenic properties. Immune-mediated liver damage induced by immunomodulatory therapies belongs to this subclass of DILI. This type of hepatotoxicity is not reproducible in animal models (4). Therefore, enhancing the understanding of immune cell-hepatocyte interplay may aid the development of reliable *in vitro* models to assess the immune-mediated hepatotoxic potential of immune-modulatory molecules (6). Various immune cell types are present in the healthy liver, including T and B lymphocytes, natural killer (NK) and NKT cells, and the myeloid dendritic cells and macrophages (7). Kupffer cells are the major tissue-resident macrophage population and have a variety of functions in the healthy liver, while also being linked to liver disease (8, 9).

Recombinant human IL-2 (rhIL-2, aldesleukin, Proleukin^®^) received marketing authorisation in the 1990s for the treatment of renal cell carcinoma and malignant melanoma (10). It is given at high doses to boost the immune response against cancer cells (11). However, aldesleukin has more functions in immunotherapy, depending on the dose. At low doses, it preferentially activates regulatory T (T_reg_) cells and is administered, for instance, to treat immune-mediated disorders. While low dose treatment is well tolerated (12), administration of high dose aldesleukin can lead to a variety of adverse events, including hepatotoxicity (10, 13). Grade 4 hepatic dysfunction, measured by bilirubin and transaminase level increases, can be observed in 11% of renal cell carcinoma patients upon high dose aldesleukin therapy (14). Management of severe toxicities includes halting the the therapy. As liver damage is reversible, results generally normalise after 5 to 6 days (15).

**Figure 1:**
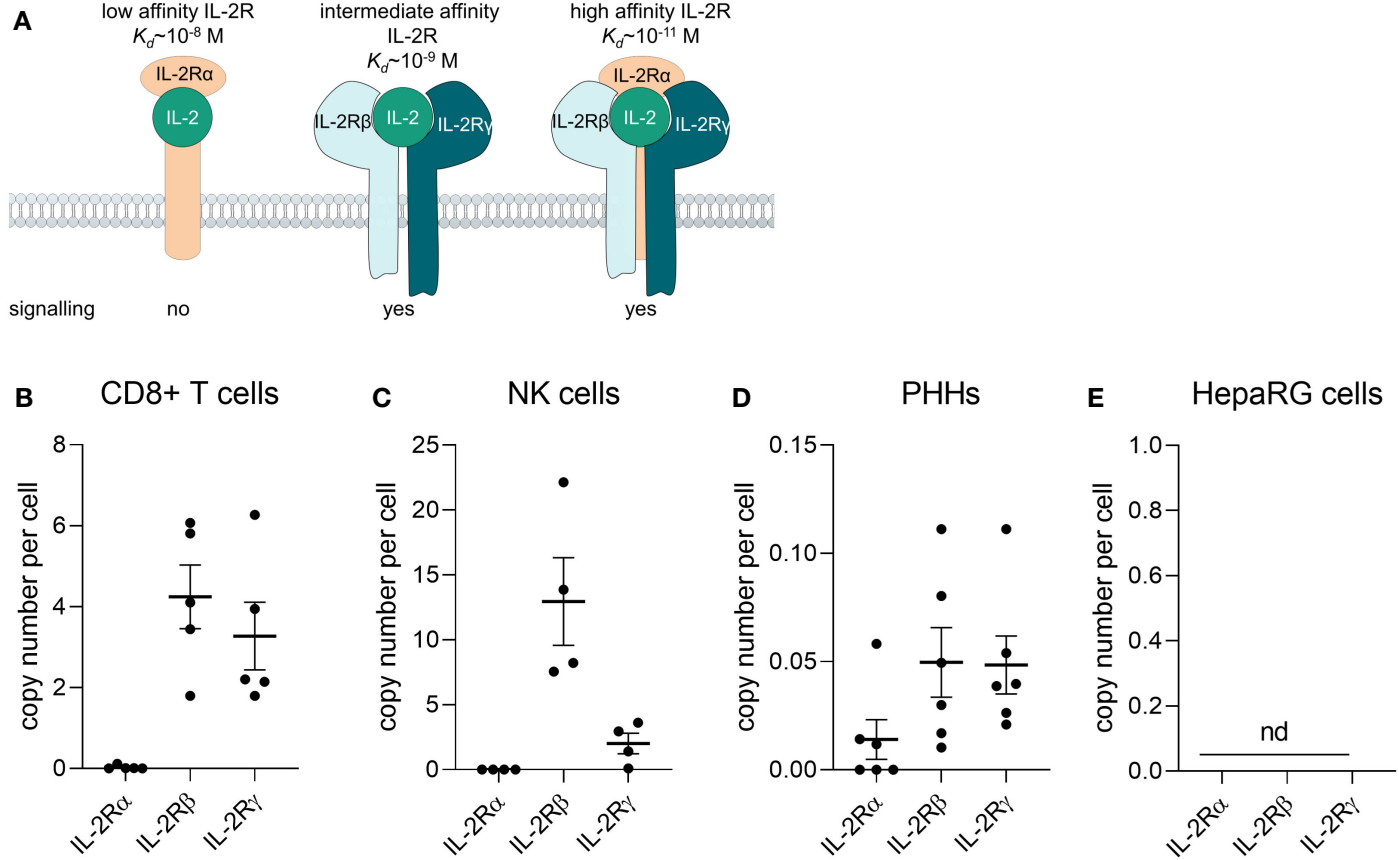
Primary human CD8^+^ T cells and NK cells express the IL-2 receptor βγ. **(A)** Composition of the IL-2 receptor (IL-2R). The IL-2Rα subunit (CD25) alone presents the low-affinity receptor, which lacks signalling capacity du to missing intracellular signalling domains. The intermediate-affinity receptor consists of the IL-2Rβ (CD122) and -γ (CD132) chains and the high-affinity receptor is consitituted of all three subunits. Adapted from (16). **(B-E)** The mRNA of IL-2Rα, -β, and –γ was quantified via qPCR **(B)** in freshly isolated CD8^+^ T cells from buffy coats (*N* = 5), **(C)** freshly isolated NK cells (*N* = 4), **(D)** primary human hepatocytes (*N* = 6) and **(E)** the HepaRG cell line (*N* = 5). Data are shown as mean ± SEM. QPCR reactions were prepared in technical duplicates and no statistical analyses was performed. NK, natural killer cells; PHHs, primary human hepatocytes; nd, not detectable.

Aldesleukin binds to the IL-2 receptor (IL-2R) which consists of up to three subunits, IL-2Rα, -β, and -γ, depending on the cell type. The IL−2Rα subunit (CD25) alone presents the low-affinity receptor, which lacks signalling capacity du to missing intracellular signalling domains. The intermediate-affinity receptor consists of the IL−2Rβ (CD122) and −γ (CD132) chains and the high-affinity receptor is constituted of all three subunits. Activation of the intermediate or high−affinity IL−2R results in JAK–STAT, ERK and PI3K signalling (**Figure 1A**) (16). The high-affinity IL-2R can for instance be found on T_reg_ cells and activated T cells (17), IL-2Rβ can be found on NK cells and IL-2Rγ on monocytes, whereby the expression level appears to be donor-dependent (18).

The exact mechanism of aldesleukin-induced hepatotoxicity remains elusive, however both direct and idiosyncratic DILI are unlikely and immune-mediated mechanisms are proposed for this drug (10). Mechanistically, leukocyte, platelet, and neutrophil recruitment to the liver in high dose aldesleukin-treated mice has been shown (19) and might also occur in humans, as ultrasound analyses suggest hepatic oedema or infiltrative processes (20). There are multiple immune cell types that might be implicated and which are therefore, addressed in this study. CD8^+^ T and NK cells are cytotoxic cells appearing abundantly in the liver. Even in the healthy state, intrahepatic lymphocytes exhibit an activated phenotype (21), have been attributed to drug-induced liver damage (22) and are not only activated by IL-2 (23, 24) but are presumably also among the main targets of high dose aldesleukin therapy (25). Activated CD8^+^ T cells induce apoptosis via FasL-Fas interaction with hepatocytes in an antigen-independent mechanism called collateral damage (26–28). In this context, IL-2 induces NK cell cytotoxicity against the hepatocellular cancer cell line HepG2 via a TRAIL-mediated mechanism (29). CD4^+^ T cell subtypes play various roles in autoimmune liver diseases (30). As such, an imbalanced T helper (Th)1/Th2 ratio has been reported to be linked to hepatotoxicity (31) and IL-2 promotes the differentiation of these cell types (32). Monocytes and monocyte-derived macrophages (MdMs) might support the mode of action of aldesleukin cancer therapy (25) and presumably play a role in the immune-mediated hepatic toxicities of immunomodulatory checkpoint inhibitor cancer therapy (33).

High dose aldesleukin provides the human body with excess rhIL-2 to promote effector cell function (11). We hypothesize that this surplus contributes to immune-mediated adverse events by overcoming tolerance induction by T_regs_ in respective tissues. This is exemplified by the case of a patient with IL-2Rα deficiency, who presented with autoimmune primary biliary fibrosis and a liver biopsy showed lymphocytic infiltration. This was interpreted to be a consequence of a decreased T_reg_ compartment and the development of autoreactive T cells to increased anti-apoptotic protein expression (34). This is also exemplified by murine knockout studies, in which functional inhibition of T_reg_ cells and concomitant low-dose IL-2 treatment replicates the toxicities observed in wild type animals with high-dose IL-2 therapy (35).

In the present study, we developed immuno-inflammatory *in vitro* hepatotoxicity test systems as models for immune-mediated DILI. We developed these models based on aldesleukin for several reasons: firstly, while the hepatotoxicity mechanism remains elusive, an immune-mediated mechanisms is proposed and there is extensive literature available on the molecular pathways evoked by rhIL-2, enabling the choice of cell types and readouts for the establishment of a novel assay. Secondly, development of a model to reflect immune-mediated hepatotoxic effects of aldesleukin may be translatable to rhIL-2 combination therapies or to novel compounds currently under development with a similar mode of action. Such recent therapy approaches include the combination of aldesleukin with checkpoint inhibitor therapy (36) or combination with cytostatic agents (ClinicalTrials.gov Identifier: NCT01038778, NCT00553618). Moreover, modified rhIL-2 molecules are being developed in order to enhance selectivity towards the intermediate affinity IL-2R (37, 38), to ameliorate pharmacokinetics by increasing the half-life (39) or to enable targeted delivery of the molecule (40).

We provide an overview of a rational approach to develop immune-mediated DILI test systems. First, we identified potential key cellular players in aldesleukin-induced immune-mediated hepatotoxicity from the literature and by analysis of drug target mRNA expression in these cell types. Secondly, we determined the effect of the drug on isolated primary human immune cell types. Then, a simple co-culture model comprised of a single immune cell type with the hepatic cell line HepaRG was established. Effects on released protein, protein surface expression and hepatocyte function were determined. The most relevant model, a direct co-culture of CD8^+^ T cells with HepaRG cells, was then re-created by using CD8^+^ T cells and primary human hepatocytes (PHHs). In-depth analysis of the inflammatory proteome was performed. Based on the results, the initial CD8^+^ T cell/HepaRG model was refined by addition of a further innate immune cell type, MdMs to the co-culture which enhanced the previously observed effects.

## 2 Methods

### 2.1 HepaRG cell culture

Undifferentiated HepaRG cells were obtained from Biopredic, France with a Material Transfer Agreement. Undifferentiated HepaRGs were cultured in Williams E medium supplemented with 10% FCS, 1% penicillin/streptomycin, 2 mM L-glutamine (all from Gibco™, Thermo Fisher Scientific, Oberhausen, Germany), 24.7 µg/mL hydrocortisone (Sigma-Aldrich, Schnellendorf, Germany) and 5 µg/mL human insulin (Lilly Deutschland, Bad Homburg, Germany). Medium was exchanged every 2-3 days and cells were passaged to reach confluency within 1 week of cell culture. Differentiation of HepaRG cells was conducted by culturing the cells for 2 weeks in the absence of DMSO, followed by cultivation for an additional 2 weeks in the presence of 2% DMSO (Sigma-Aldrich, Schnellendorf, Germany), according to a published protocol (41). HepaRGs were used to a maximum passage number of 20. All cells were cultured at 37°C in a 5% CO_2_ atmosphere.

### 2.2 Primary human hepatocytes

PHHs were isolated and cryopreserved from resected liver tissues as previously published (42) and were provided by Dr. Georg Damm (University Hospital, Leipzig University Leipzig, Germany). All patients gave their informed consent for the usage of their biomaterials for research purposes according to the ethical guidelines of the Medical Faculty of Leipzig University (Ethical vote: registration number 322/17-ek, date 2020/06/10 ratified on 2021/11/30 and registration number 238/22-ek, date 2022/07/18). Thawing and cultivation procedures were adapted from Keeminck et al. (43), with the kind support of Dr. Andrea Scheffschick (University Hospital, Leipzig University Leipzig, Germany). Briefly, PHHs were thawed in Williams E Medium supplemented with 10% FBS, 15 mM HEPES, 1 mM sodium pyruvate, 1% MEM NEAA and 1% penicillin/streptomycin, 1% L-Glutamine (all from Gibco™, Thermo Fisher Scientific, Oberhausen, Germany), 32 IU/L human insulin and 1 µg/mL dexamethasone (Sigma-Aldrich, Schnellendorf, Germany). Cell viability was determined using the Trypan-blue (Gibco™, Thermo Fisher Scientific, Oberhausen, Germany) exclusion technique. Cell viability in all experiments was >80%. Cells were seeded onto 96-well plates which had been coated over night with rat-tail collagen (20 µg/mL) which was kindly provided by Fraunhofer ISC, Würzburg, Germany. Cell density was 80,000 PHHs per well. After 24 h incubation at 37°C in a 5% CO_2_ atmosphere, cell layers were washed twice with DPBS, calcium, magnesium from Gibco™ (Thermo Fisher Scientific, Oberhausen, Germany). A sandwich culture was created by overlay with 0.25 mg/mL Geltrex™ (Gibco™, Thermo Fisher Scientific, Oberhausen, Germany). PHH culture was performed with Williams E Medium supplemented with 1% penicillin/streptomycin, 1% L-Glutamine, 1% insulin-transferrin-selenium pre-mix (Gibco™, Thermo Fisher Scientific, Oberhausen, Germany) and 0.1 M dexamethasone. Medium was replaced daily, except over the weekend, when medium was replaced every other day. At day 3 or 6 of culture, cells were taken into co-culture experiments (see below).

### 2.3 PBMC isolation

PBMCs were isolated from buffy coats via density gradient as described previously (44). Briefly, 25 mL of heathy donor blood (German Red Cross, Frankfurt, Germany) was mixed with an equal volume of HBSS (Thermo Fisher Scientific, Oberhausen, Germany) and layered over 15 mL of Biocoll (Merck, Darmstadt, Germany) in Sep-Mate™-15 tubes (Stemcell Technologies, Cologne, Germany). After centrifugation (1,200 g, 10 min, RT), PBMCs were collected from the interphase. Cells were washed with 2 mM EDTA/PBS for 3 times and counted in a MACSQuant^®^ Analyzer 10 flow cytometer (Miltenyi Biotec, Bergisch Gladbach, Germany).

### 2.4 Isolation, expansion and polarization of primary human immune cell subtypes

All kits for immune cell isolation were purchased from Miltenyi Biotec (Bergisch Gladbach, Germany) and immune cell subtypes were freshly isolated from PBMCs. CD8^+^ T cells were isolated via positive selection with human CD8 MicroBeads, NK cells were isolated by negative selection using the Human NK cell Isolation Kit, naïve CD4^+^ T cells were isolated via negative selection with the human Naive CD4^+^ T Cell Isolation Kit II, T_reg_ cells were isolated with the CD4^+^CD25^+^ Regulatory T Cell Isolation Kit by depletion of non-CD4^+^ T cells and positive selection of CD25^+^ cells, and CD14^+^ monocytes were isolated via positive selection with Human CD14 MicroBeads. Neutrophils were isolated directly from buffy coats via double gradient centrifugation as previously described (45). When cell culturing was needed, primary human immune cells were seeded in X-Vivo-15 medium (Lonza, Basel, Switzerland). CD8^+^ T cells were used directly in experiments or pre-cultivated for 7 d in the presence of 50 IU/mL rhIL-2 (Biolegend, Amsterdam, Netherlands). Except for the baseline IL-2R mRNA expression qPCR, NK cells were expanded according to the manufacturers’ protocol with the human NK Cell Activation/Expansion Kit (Miltenyi Biotec, Bergisch Gladbach, Germany) and 500 IU/mL rhIL-2. NK cells were expanded for 11 or 12 days prior to application in the experiments. Naïve CD4^+^ T cells were differentiated into Th1 and Th2 subtypes as previously described (46). Briefly, naïve CD4^+^ T cells were activated for 4 days with plate-bound anti-CD3 antibody (2 µg/mL), soluble anti-CD28 antibody (2 µg/mL), and rhIL-2 (50 IU/mL). For polarization towards the Th1 cell subtype, additionally rhIL-12 (2.5 ng/mL) and anti-IL-4 antibody (5 µg/mL) were added, for Th2 polarization, rhIL-4 (12.5 ng/mL), anti-IFNγ antibody (5 µg/mL) and anti-IL-10 antibody (5 µg/mL) were added. All antibodies and cytokines for the Th1/Th2 polarization were purchased from Biolegend (Amsterdam, Netherlands) with the exception of the anti-CD28 antibody which was acquired from Miltenyi Biotec (Bergisch Gladbach, Germany). After 4 days incubation, medium was exchanged with medium containing the respective stimuli but in the absence of anti-CD3 and anti-CD28 antibodies. On day 8 or day 14, cells were harvested and applied in the mono-culture experiments. T_reg_ cells were expanded for 14 d according to the manufacturer’s protocol using the T_reg_ Expansion Kit (Miltenyi Biotec, Bergisch Gladbach, Germany) and 500 IU/mL rhIL-2. CD14^+^ monocytes were polarized to MdMs via addition of rhGM-CSF (f.c. 10 ng/mL, Miltenyi Biotec, Bergisch Gladbach, Germany) for 7 d without medium change.

### 2.5 Stimulation of primary human immune cell subtypes

Primary human CD8^+^ T, NK, Th1 or Th2 cells were stimulated with aldesleukin (Proleukin^®^ S, Clinigen, Burton upon Trent, Great Britain) in the ranges of 1 to 5000 IU/mL. As controls, immune cells were stimulated with vehicle (PBS/BSA) or with activation controls. For CD8^+^ T and Th cell subtypes, cells were stimulated with anti-CD3/anti-CD28 beads (bead-to-cell ratio of 4:1; Miltenyi Biotec, Bergisch Gladbach, Germany) and 50 IU/mL rhIL-2 or with Phytohemagglutinin-L (PHA-L, f.c. 2.5 µg/mL; Thermo Fisher Scientific, Waltham, USA) and 50 IU/mL rhIL-2. NK cells were activated with the human NK Cell Activation/Expansion Kit (anti-CD2, anti-CD335(NKp46), Miltenyi Biotec, Bergisch Gladbach, Germany) and 500 IU/mL rhIL-2 or with the human NK Cell Activation/Expansion Kit and rhIL-15 (f.c. 10 ng/mL, Biolegend, Amsterdam, Netherlands) and rhIL-21 (f.c. 25 µg/mL, Biolegend, Amsterdam, Netherlands). For the immune cell mono-culture assays, CD8^+^ T and Th cell subtype stimulation was conducted for 5 d, NK cell stimulation occurred for 2 d. Subsequently, immune cells were analysed by flow cytometry or qPCR analyses.

### 2.6 Primary human immune cell/HepaRG co-culture

The co-culture and triple co-culture assays with primary human immune cells and hepatocytes were performed in a 96-well format. HepaRGs were seeded in a density of 9000 cells per well of a 96-well plate, when differentiated and after reaching confluency, the approximate cell number was 40,000 HepaRGs per well. For the co-cultures of primary human CD8^+^ T or NK cells with HepaRG cells, the immune cells were stimulated as described above but incubation times were adapted to reach a total assay time of 5 d or 2 d, respectively. For the indirect co-culture, HepaRG cells were cultivated in the lower chamber of 96-well HTS Transwell^®^ plates with a pore size of 0.4 µM (Corning, New York, USA). For cytotoxicity assessment, freshly stimulated CD8^+^ T cells (0.1*10^6^ cells/well) were co-incubated with HepaRG cells for 5 d. For functional assays, 0.1*10^6^ CD8^+^ T cells/well were pre-incubated with aldesleukin or activation controls as described above for 2 d, prior to 3 d co-culture with HepaRG cells. For indirect NK co-cultures, 0.1*10^6^ freshly stimulated NK cells were added into the upper chambers of the transwell plate and co-cultured with HepaRG cells for 2 d. For the direct co-cultures, a co-culture medium of 50% v/v X-Vivo-15 medium and 50% v/v HepaRG differentiation medium was used. Immune cells were stimulated in this medium. CD8^+^ T cell cytotoxicity was determined by addition of 0.1*10^6^ CD8^+^ T cells on top of the HepaRG cells for 5 d. For functional assays, 0.1*10^6^ CD8^+^ T cells were pre-stimulated for 3 d and then the cell suspension was added on top of the HepaRG cells for additional 2 d. For the direct NK cell/HepaRG co-culture, 0.1*10^6^ NK cells were pre-incubated with the stimuli for 44.5 h before adding them on top of the HepaRG cells for 3.5 h. For the triple co-cultures, 0.1*10^6^ CD8^+^ T cells were co-incubated with 0.025*10^6^ MdMs under aldesleukin stimulation or the described activation stimuli for 3 d in 96-well U-bottom plates before addition onto HepaRG cells for 2 d for functional assays or the CD8+ T cell/MdM mixture was added directly after stimulation onto HepaRG cells for 5 d for the analysis of cytotoxicity.

### 2.7 Primary human CD8^+^ T cell/PHH co-culture

For the direct co-culture of CD8^+^ T cells with PHHs, a co-culture medium of 50% X-Vivo-15 medium and 50% PHH culture medium was used. CD8^+^ T cells were stimulated in this medium and cytotoxicity was determined by addition of 0.1*10^6^ CD8^+^ T cells on top of PHH cells in sandwich culture (at day 3 after seeding) for 5 d. For functional assays, 0.1*10^6^ CD8^+^ T cells were pre-stimulated for 3 d and then added onto PHHs in sandwich culture (at day 6 after seeding) for additional 2 d.

### 2.8 HepaRG cytolysis assay

The cytolytic activity of primary human NK cells towards HepaRG cells was determined in a cytolysis assay. Single cell suspensions of differentiated HepaRG cells were stained with CellTrace™ Violet (Thermo Fisher Scientific, Waltham, USA). To a 96-well U-bottom plate, 0.125*10^6^ NK cells and 0.025*10^6^ HepaRG cells were seeded (effector:target ratio of 5:1). To accumulate the cells in the centre of the wells, plate was pulsed in a centrifuge for 10 sec. After 6 h incubation (37°C, 5% CO_2_), cells were stained with 7-AAD (BD, Heidelberg, Germany) followed by acquisition in the MACSQuant^®^ Analyzer 10. HepaRG cells were identified by CellTrace™ Violet stain and the percentage of 7AAD^+^ (dead) HepaRG cells was determined in the FlowJo 10.5.3 software (BD, Heidelberg, Germany).

### 2.9 LDH assay

The release of lactate dehydrogenase (LDH) into the supernatant was measured with the Cytotoxicity Detection Kit^PLUS^ (Roche Holding, Basel, Switzerland). As positive (lysis) control, HepaRG cells or PHHs were lysed with 10 µL of lysis solution for 6 h. As background control, medium was analysed. Supernatant from samples and controls was collected, centrifuged and analysed according to the instructions of the manufacturer. Samples and controls were analysed in duplicates. The mean values of all samples and controls were calculated. The background control value was subtracted from the samples and the lysis control. Sample values were related to the lysis control and expressed as percentage of lysis control.

### 2.10 CRP Enzyme-linked immunosorbent assay

CRP concentrations in the cell culture supernatants were quantified with human ELISA kit according to the protocols of the manufacturer (Thermo Fisher Scientific, Oberhausen, Germany).

### 2.11 Quantitative real-time PCR

The expression of the IL-2R subunits as well cytokines and polarisation markers IFN-γ, T-bet, GATA3, IL-4, FOXP3, Eomes, and RoRγC were measured in qPCR experiments (**Supplementary Table 1**). Total RNA was isolated with the RNeasy^®^ Plus Mini kit with a DNA digestion step performed with the RNase-Free DNase Set (both kits from Qiagen, Hilden, Germany). The RNA was reverse transcribed into cDNA with the First strand cDNA synthesis kit (Thermo Fisher Scientific, Oberhausen, Germany). Amplification of cDNA was performed and quantified in the CFX96 Touch Real-Time PCR Detection System (Bio-Rad Laboratories, Inc., Hercules, USA) using the 5x qPCR Mix EvaGreen (Rox, Bio&SELL, Feucht, Germany). Primers were applied at a final concentration of 0.5 µM and synthesized by biomers.net GmbH (Ulm, Germany). For the quantification of IL-2R subunit transcript numbers, a standard curve for the genes of interest IL-2Rα, -β, -γ and the reference genes βActin and TBP-1 was generated, allowing for back-calculation of numbers of IL-2R subunit transcripts. For the analysis of the polarisation marker, the ΔΔCT method was used and the expression levels were normalised to the expression of the housekeeping genes βActin and TBP-1. Data were analysed in the BioRad CFX Manager 3.1 Software (Bio-Rad Laboratories, Inc.).

### 2.12 Flow cytometry analyses

Surface marker expression on immune cells and hepatocytes was analysed by flow cytometry. Live/dead cell discrimination was addressed with Zombie Violet staining (Biolegend, Amsterdam, Netherlands) in immune cells and in hepatocytes via 7-AAD. For surface marker analyses, the following anti-human antibodies were acquired from Miltenyi Biotec (Bergisch Gladbach, Germany): CD4-VioGreen, CD8-VioGreen, CD16-PE, CD16-PE-Vio615, CD18-PE-Vio770, CD25-APC-Vio770, CD49A-APC-Vio770, CD54-APC, CD69-PE-Vio770, CD95-PE, CD122-APC, CD158d-PE, CD178-PE, CD178-PE-Vio770, CD192-APC-Vio770, CD253-APC, CD314-PE-Vio770, HLA Class I B8-PE-Vio770, HLA-DR-APC-Vio770. The following anti-human antibodies were obtained from Biolegend (Amsterdam, Netherlands): CD56-BV510, CD40-Pacific Blue, CD262-APC. From BD (Heidelberg, Germany), the anti-human antibodies CD132-BB700, CD314-BV421 and CD261-BV510 as well as Annexin V- PE were purchased. An overview over the markers measured per cell type can be found in **Supplementary Table 2**. All flow cytometry analyses were conducted immediately after the indicated respective incubation and/or co-culture time points. To analyse immune cell viability and apoptosis, the lymphocyte population was gated and single cells were determined. Co-staining of Annexin V and Zombie Violet was used for the discrimination of viable (Zombie Violet^-^/Annexin V^-^), early apoptotic (Zombie Violet^-^/Annexin V^+^) and late apoptotic (Zombie Violet^+^/Annexin V^+^) cells. The cell numbers in the respective fractions were related to the total cell number of all three fractions and expressed as percent. For surface marker expression analyses in immune cells, the lymphocyte population was gated, then single cells were selected and dead cells were excluded. Then, the target cell population was identified. For hepatocytes, debris was excluded, single cells were selected and dead cells were excluded from analyses. PHH were difficult in the flow cytometric analysis due to their sensitivity to sheer stress.

For the quantification of cytokines in the cell culture supernatant, the MACSPlex Cytotoxic T/NK Cell Kit, human (Miltenyi Biotec, Bergisch Gladbach, Germany) was used according to the manufacturer’s protocol.

### 2.13 Olink protein analysis

Protein analysis of 92 human immune system and inflammation-related proteins was performed using the Olink^®^ Target 96 inflammation assay (Olink, Uppsala, Sweden). 1 µL of 1:3 diluted supernatants of direct CD8^+^ T cell/PHH co-cultures was measured. Plate loading was randomized and the samples blinded. Quality control of the run was conducted following the supplier’s specifications. The Olink protein analysis relies on the proximity extension assay technique that allows for the simultaneous detection of 92 proteins in one sample (47). Resulting data are given as an arbitrary unit (NPX, Normalized Protein eXpression) which is on a log2 scale.

### 2.14 Heatmap analysis via R

To give an overview over co-culture readouts, R version 4.2.0 (2022-04-22 ucrt) was employed. The median values of the readouts were normalised to a 0 to 1 scale and analysed in a heatmap using the packages “heatmaply” and “pheatmap”. Similarly regulated readouts were clustered using the Ward’s minimum variance method.

### 2.15 Correlation analysis via R

Correlation of hepatocyte marker and cytokine expression as well as cytotoxicity from direct CD8^+^ T/HepaRG cell, CD8^+^ T/PHH and CD8^+^ T/MdM/HepaRG cell co-cultures was conducted using the R packages “corrplot” and “Hmisc”. For each co-culture model the median values for the readouts were calculated and analysis was performed with aldesleukin stimulated samples, but not with activation control in order to elucidate relevant correlations for aldesleukin data. Only direct co-cultures were implied as only in these co-cultures, aldesleukin-mediated cytotoxicity effects were observed. p < 0.05 was considered the threshold for significance.

### 2.16 Statistics

GraphPad Prism 8 (GraphPad, San Diego, USA) was applied for the calculations and for the creation of graphs. Results are shown as mean ± standard error of the mean (SEM). The numbers of independently performed experiments (*N*) and the number of replicates per experiment are stated in the corresponding figure captions. Outliers were not removed, unless otherwise indicated in the figure captions. The data were analysed for normal distribution via Shapiro-Wilk test and analysis of the QQ plot. According to distribution of the groups, significant differences were determined with the parametric one-way ANOVA with Dunnett’s correction for multiple comparisons or with the non-parametric Kruskal-Wallis Test with Dunn´s correction. Where indicated, two-way ANOVA with Dunnett´s correction was applied. p < 0.05 was considered the threshold for significance. If not otherwise indicated, significant differences between aldesleukin-treated or activated samples and vehicle-treated samples were determined.

## 3 Results

### 3.1 Primary human CD8^+^ T and NK cells expressed the intermediate-affinity IL-2R

To identify which cell types are potentially responsive to aldesleukin, the mRNA expression of the IL-2R subunits α, β and γ was assessed in various primary immune cells (e.g. CD8^+^ T cells, monocytes, MdMs, Th1 and 2 cells, T_reg_ cells, NK cells, neutrophils) by two-step qPCR. In freshly isolated CD8^+^ T cells and NK cells, the IL-2Rβ and IL-2Rγ mRNA could be detected, whereas the IL-2Rα mRNA was close to the detection limit (**Figures 1B, C**). Expression of the intermediate affinity IL-2R (β/γ) in these cell types is in line with the literature (18, 48). Th1, Th2 and T_reg_ cells expressed the trimeric IL-2R. CD14^+^ monocytes, MdMs and neutrophils expressed IL-2Rγ to different extents, with the most pronounced expression in MdMs (**Supplementary Figure 1A**). For aldesleukin signalling, the dimeric (β/γ) or trimeric (α/β/γ) IL-2R is necessary (49). The effect of aldesleukin stimulation on IL-2R expression was investigated in CD8^+^ T, Th1, Th2 and NK cells, as those cell types, due to their IL-2R expression profile, are generally capable of inducing an intracellular signalling response. We tested aldesleukin concentrations from 1 to 5000 IU/mL. Concentrations up to 1000 IU/mL represent the range in which aldesleukin can be detected in human serum after treatment (50) and 5000 IU/mL was tested as in the *in vitro* setting, it is often necessary to exceed *in vivo* blood concentrations to identify toxic drugs (51). Mono-cultures of T cell subtypes were stimulated with aldesleukin for 5 d, NK cells were stimulated for 2 d. With the exception of increased IL-2Rαβ levels in NK cells, no significant impact of aldesleukin stimulation on IL-2R subunit mRNA expression in the other immune cell types was found (**Supplementary Figures 1B-E**), indicating that aldesleukin increases affinity of NK cells towards this molecule.

We were interested in the IL-2R expression on HepaRG cells and primary human hepatocytes (PHHs) to investigate potential direct effects of aldesleukin on hepatocytes. In HepaRGs, no IL-2R mRNA expression above the lower limit of detection was measurable. PHHs expressed low IL-2R mRNA levels close to the detection limit, probably due to residual hepatic non-parenchymal cells in the samples. This contamination might arise from the PHH isolation process which is based on density-gradient centrifugation and does not allow to obtain a 100% pure PHH fraction (52). This suggests a low or absent responsiveness of HepaRG cells or PHHs towards aldesleukin (**Figures 1D, E**).

### 3.2 Aldesleukin upregulated TRAIL and adhesion molecule expression in primary human CD8^+^ T and NK cells

**Figure 2:**
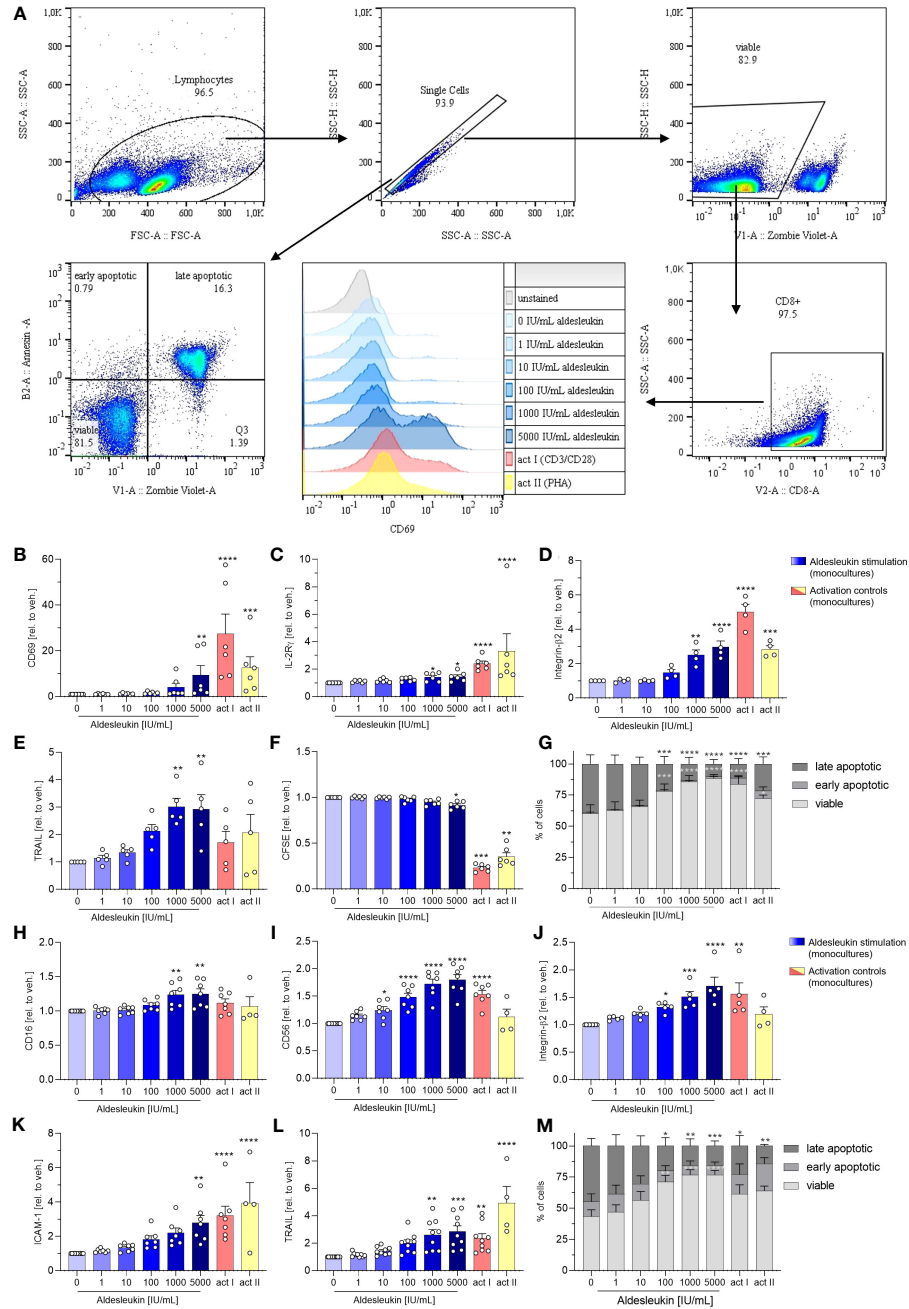
Aldesleukin increases primary human CD8^+^ T and NK cell cytotoxicity and adhesion marker expression. **(A)** Gating strategy for surface marker analysis and determination of fractions of viable, early apoptotic and late apoptotic cells. Representative plots from one CD8^+^ T cell monoculture experiment are shown. **(B-G)** Primary human CD8^+^ T cells were stimulated for 5 d with aldesleukin or activation controls (act I, CD3/CD28 + rhIL-2 activation; act II, PHA-L + rhIL-2 activation). **(H-M)** Primary human NK cells were stimulated for 2 d with aldesleukin or activation controls (act I, CD2/CD335 + rhIL-2 activation; act II, CD2/CD335 + rhIL-15/21 activation). Per stimulation point, 0.1 x 10^6^ cells were stimulated. To quantify surface marker expression, cells were analysed by flow cytometry and the mean fluorescence intensity was related to the vehicle control. The fractions of viable (Zombie Violet^-^/Annexin V^-^), early apoptotic (Zombie Violet^-^/Annexin V^+^), and late apoptotic (Zombie Violet^+^/Annexin V^+^) cells were determined as described in the methods section. Number of biological replicates: *N* = 3 **(G)**; *N* = 4 **(D, M)**; *N* = 5 **(E, J)**; *N* = 6 **(B, C, F)**. *N* = 7 **(H, I, K)**; *N* = 9 **(L)**. In some cases, there are fewer biological replicates for the activation controls. One technical replicate was performed except for CFSE dilution assays. Data are shown as mean ± SEM. For statistical analysis, one-way ANOVA with Dunnett´s correction **(D, E, F, I, K, L)**, Kruskal Wallis test with Dunn´s multiple comparison test **(B, C, H, J)** or two-way ANOVA with Dunnett´s correction **(G, M)**, was used. * p < 0.05, ** p < 0.01, *** p < 0.001 and **** p < 0.0001 indicate significant differences between aldesleukin-treated or activated and vehicle-treated samples. CFSE, Carboxyfluorescein succinimidyl ester; ICAM-1, intercellular adhesion molecule 1; TRAIL, Tumor Necrosis Factor Related Apoptosis Inducing Ligand.

Next, we analysed the direct effects of aldesleukin on primary human immune cells. We addressed CD8^+^ T, Th1, Th2 and NK cells, as they express IL-2R mRNA. Furthermore, CD8^+^ T and NK cells might be implicated in immune-mediated hepatotoxicity as they are cytotoxic cells which are abundant in human liver (21), have been attributed to drug-induced liver damage (22) and are activatable by IL-2 (23, 24). Moreover, Th1 and Th2 cells might be crucial for liver toxicity, as IL-2 can promote the differentiation of these cell types (32) and Th1/Th2 imbalance has been shown to be linked to hepatotoxicity (31). The impact of aldesleukin on immune cell mono-cultures was assessed by the detection of several surface markers related to activation, adhesion and cytotoxicity via flow cytometry (**Figures 2A** and **Supplementary Table 2**). In each experiment, two immune cell activation controls were included to capture effects of a robust activation on the readouts and to enable comparison of aldesleukin effects to activated cells. For CD8^+^ T cells, T cell receptor ligation was activated by antiCD3/antiCD28 antibodies or by the mitogen PHA-L. NK cells were activated by both rhIL-2 and anti-CD2/anti-CD335 antibodies or by rhIL-15/rhIL-21 with anti-CD2/anti-CD335 antibodies.

High aldesleukin concentrations upregulated the surface expression of the activation marker CD69, IL-2Rγ, integrin -β2, and the apoptosis-inducing ligand TRAIL concentration-dependently (**Figures 2B–E**). 5000 IU/mL aldesleukin slightly increased proliferation and concentration-dependently increased viability of CD8^+^ T cells (**Figures 2F, G**). Overall, the highest aldesleukin concentrations regulated surface markers in similar directions to the activation controls, however the CD3/CD28 activation produced a more pronounced response in most markers. In contrast, TRAIL surface expression was only increased by aldesleukin and not by the activation controls. PHA-L stimulation is reported to induce soluble TRAIL and not the membrane bound form (53) which was detected in flow cytometry analyses, providing an explanation for the lack of TRAIL induction in the activation controls. Aldesleukin had no impact on surface marker expression of integrin-α1, IL-2Rαβ or Fas ligand (**Supplementary Figures 2A-D**).

In NK cells, the expression of the activation marker CD16 and the NK cell lineage marker CD56 were concentration-dependently enhanced by aldesleukin (**Figures 2H, I**). CD16 was not increased by the activation controls and CD56 was not induced by activation control II (CD2/CD335 + rhIL-15/21), indicating that aldesleukin has a different impact on NK cell polarisation than do the activation controls. In terms of polarization, it should be considered that NK cells were expanded prior to the experiment leading to increased CD56 expression (**Supplementary Figure 3**) and high CD56 promotes NK cell responsiveness to IL-2 (54). Further, aldesleukin concentration-dependent integrin-β2, ICAM-1 and TRAIL upregulation was measured as well as increased viability (**Figures 2J–M**). The activation control I (CD3/CD28 + rhIL-2) regulated these markers in a similar manner to high aldesleukin concentration. Aldesleukin did not induce proliferation or regulate surface expression of IL-2Rα, -β, -γ, activation marker NKG2D and KIR2DL4, C-C chemokine receptor type 2, Fas ligand or integrin-α1 on NK cells (**Supplementary Figures 2E-M**).

In summary, surface marker expression data point towards increased cytotoxicity and tissue-migration potential of CD8^+^ T and NK cells after aldesleukin stimulation, which are hypothesised to be important steps in the development of aldesleukin-mediated hepatotoxicity.

### 3.3 Aldesleukin had no direct effect on T helper cells and hepatocytes

In Th1 cells, aldesleukin increased IL-2Rα and CD69 surface expression, but had no impact on adhesion or cytotoxicity marker expression (**Supplementary Figures 4A-G**). In Th2 cells, of the tested surface markers only CD69 was augmented by high aldesleukin concentrations (**Supplementary Figures 4H-N**). As shifts in Th cell polarization are reported to be conducive to immune-mediated hepatotoxicity (55), we addressed Th cell polarization marker expression by two-step qPCR. Aldesleukin did not have any significant impact on Th cell polarization marker mRNA expression (IFN-γ, T-bet, GATA3, IL-4, FOXP3 or RoRγC mRNA) in Th1 (**Supplementary Figures 5A-F**) and Th2 cells (**Supplementary Figures 5G-L**).

To evaluate direct effects of aldesleukin on HepaRG cells and PHHs, the impact of 5000 IU/mL aldesleukin in comparison to CD8^+^ T cell activation controls was addressed on surface marker expression in these cell types. No immediate effects of aldesleukin on hepatocyte cell types were detected (**Supplementary Figure 6**).

### 3.4 Aldesleukin-induced hepatotoxicity in direct CD8^+^ T cell/HepaRG and NK cell/HepaRG co-cultures

**Figure 3:**
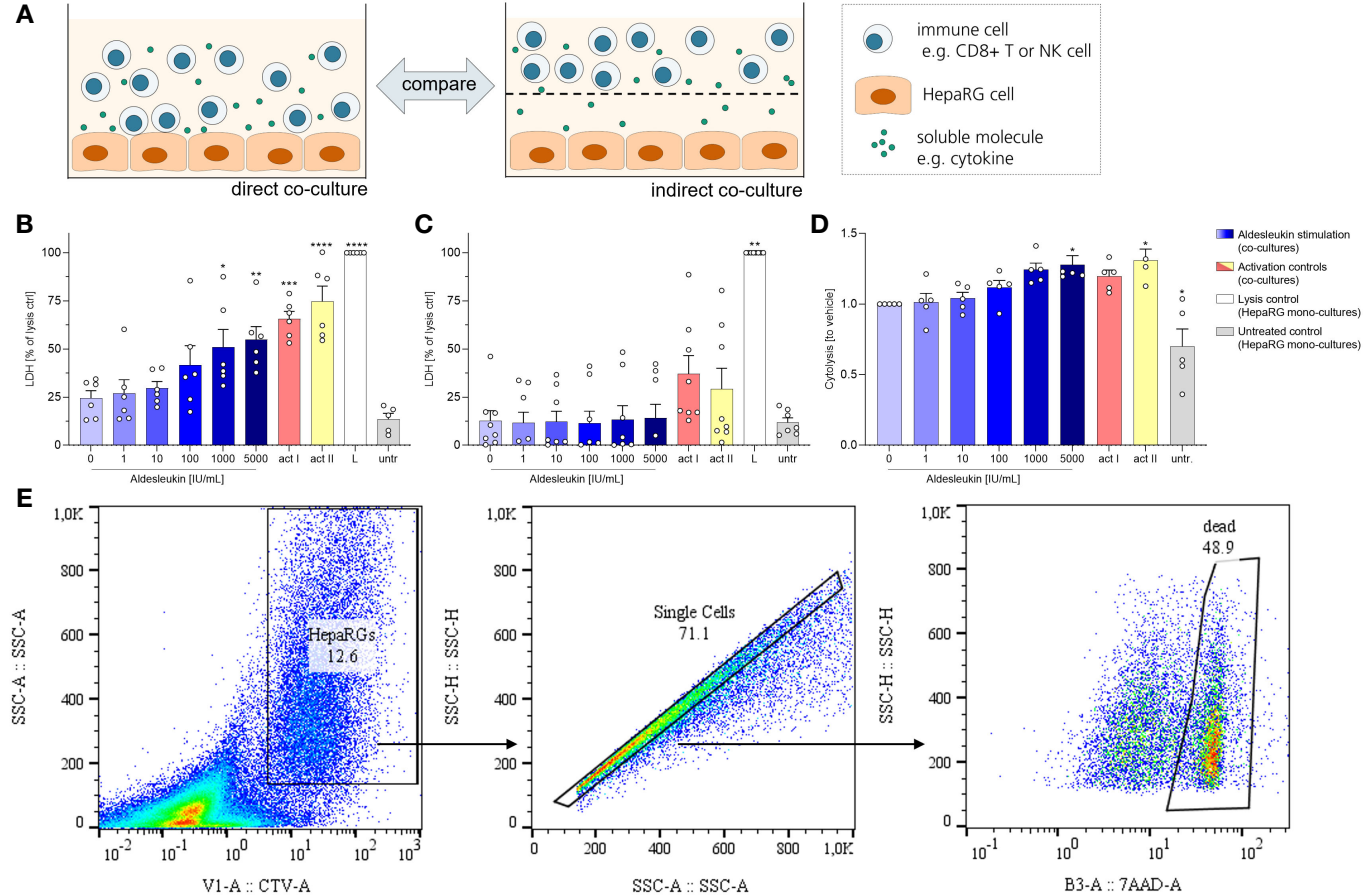
Aldesleukin increased cytotoxicity in direct immune cell/HepaRG co-cultures. **(A)** For evaluation of cytotoxic effects of immune cells towards HepaRGs, direct and indirect co-cultures were established. **(B, C)** Primary human CD8^+^ T cells were stimulated with aldesleukin or activation controls (act I, CD3/CD28 + rhIL-2 activation; act II, PHA-L+ rhIL-2 activation) and co-incubated with HepaRG cells for 5 d **(B)** in direct co–cultures or **(C)** in indirect co-cultures in which CD8^+^ T cells were separated from HepaRG cells by a transwell insert. Colorimetric assessment of lactate dehydrogenase (LDH) activity was conducted to measure cell-mediated cytotoxicity. Samples were referred to the lysis control. **(D)** Expanded primary human NK cells were pre-treated with aldesleukin or activation controls (act I, CD2/CD335 + rhIL-2 activation; act II, CD2/CD335 + rhIL-15/21 activation) for 42 h and co-cultivated with HepaRG single cell suspensions for 6 h. HepaRG cell viability was determined via flow cytometry and referred to the vehicle-treated samples. **(E)** Gating strategy for the cytolysis assay. To discriminate HepaRG cells from immune cells, HepaRG cells were stained with CellTrace™ Violet (CTV). Data are shown as mean ± SEM. *N* = 5 **(D)**, *N* = 6 **(B)**, *N* = 8 **(C)**. LDH measurements were acquired in technical duplicates, for the cytolysis assay one technical replicate was acquired. For the cytolysis assay, samples in which activation control I did not induce lysis compared to vehicle-treated samples were excluded from the analyses, as NK cells were perceived as non-activatable. For statistical analysis of **(B, D)** one-way ANOVA with Dunnett´s correction and for analysis of **(C)** Kruskal Wallis test with Dunn´s multiple comparison test was used. * p < 0.05, ** p < 0.01, *** p < 0.001 and **** p < 0.0001 indicate significant differences between aldesleukin-treated or activated and vehicle-treated samples. L, Triton-X-lysed HepaRG cells; LDH, lactate dehydrogenasse; untr., untreated HepaRG monocultures.

We next investigated the cytotoxic effects of aldesleukin-stimulated immune cells on HepaRG cells. HepaRG cells are reported to display PHH functions while providing increased reproducibility and improved accessibility compared to primary cells in hepatotoxicity assessment (56). For potential cytotoxicity, two co-culture models were tested: One allowed cell-cell contact of immune cells and hepatocytes (direct co-culture) and in another immune cells and hepatocytes were separated by a transwell insert (indirect co-culture, **Figure 3A**). As CD8^+^ T and NK cells responded to aldesleukin with increased adhesion and cytotoxicity marker expression, we used these cells for the co-culture models.

To capture cytotoxic effects of CD8^+^ T cells on hepatocytes, cells were co-cultured for 5 d following aldesleukin stimulation. 1000 and 5000 IU/mL aldesleukin significantly increased lactate dehydrogenase (LDH) release, a surrogate marker for cellular integrity, indicating a cytotoxic effect towards HepaRG cells similar to activation controls (**Figure 3B**). In contrast, when CD8^+^ T cells and HepaRGs were separated by transwell inserts, no cytotoxicity effect of aldesleukin or of the activation controls was observed (**Figure 3C**), indicating that direct cell-cell contact is mandatory for this effect. Aldesleukin had no intrinsic cytotoxic effect on hepatocytes (**Supplementary Figure 7A, B**).

We addressed NK cell cytotoxicity in a cytolysis assay, as LDH assessment proved inappropriate to determine cytotoxic effects in our NK cell models (**Supplementary Figure 7C, D**). For the cytolysis assay, NK cells were pre-stimulated with aldesleukin for 42 h and added to HepaRG target cells in suspension for 6 h. Compared to vehicle-treated samples, 5000 IU/mL aldesleukin significantly increased HepaRG cytolysis as determined by the number of dead (7AAD+) HepaRG cells (**Figure 3D, E**).

### 3.5 CD8^+^ T cell/HepaRG interactions influence hepatic surface marker expression and cytokine release at high aldesleukin concentrations

To characterize the interactions of hepatocytes and primary human CD8^+^ T cells, direct and indirect co-cultures with HepaRG cells were established. To mitigate the cytotoxic effects described above, the time of co-culture was adapted. For the direct co-culture experiments, CD8^+^ T cells were pre-stimulated for 3 d before addition to HepaRG layers for 2 d. For the indirect co-culture, CD8^+^ T cells were pre-stimulated for 2 d before addition to transwell inserts that were placed on top of HepaRG cells for 3 d. This yielded a total experiment duration for CD8^+^ T cell/HepaRG co-cultures of 5 d, ensuring comparability to the preceding mono-culture experiments.

**Figure 4:**
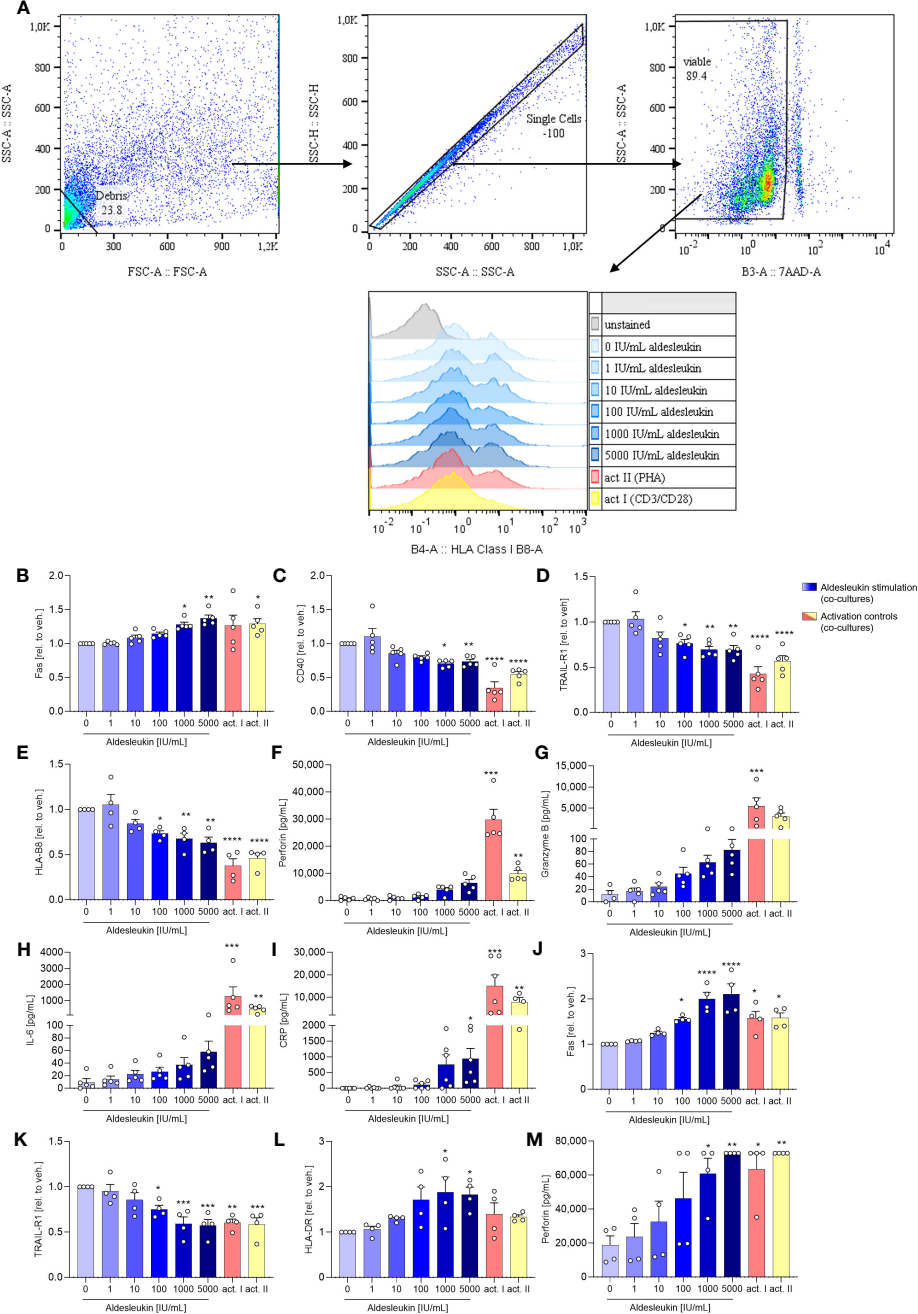
Aldesleukin influences hepatic surface marker expression and pro-inflammatory mediator release in direct co-cultures of immune cells with HepaRGs. **(A)** Flow cytometry gating strategy for surface marker detection on hepatocytes following co-culture with immune cells. Representative plots of one direct CD8^+^ T/HepaRG cell co-culture experiment are shown. Please note that in the first step, debris is excluded from analysis via a “not-gate”. **(B-I)** Primary human CD8^+^ T cells were pre-stimulated with aldesleukin or activation controls (act I, CD3/CD28 + rhIL-2 activation; act II, PHA-L+ rhIL-2 activation) for 3 days and co-cultivated with HepaRG cells for 2 days in direct co-cultures. **(J-M)** Primary human NK cells were pre-incubated with aldesleukin or activation controls (act I, CD2/CD335 + rhIL-2 activation; act II, CD2/CD335 + rhIL-15/21 activation) for 44.5 h and co-cultivated with HepaRG cells for 3.5 h. **(B-E, J-L)** Hepatic surface marker expression was determined with flow cytometry and the mean fluorescence intensity was related to the vehicle control. **(F-I, M)** Pro-inflammatory mediators were quantified via flow cytometry, CRP was determined via ELISA. Data are shown as mean ± SEM. *N* = 4 **(J-M)**, *N* = 5 **(B-L)**. For **(E)**, one outlier was removed. Samples were acquired in one technical replicate. For statistical analysis of **(C-E, G, J- L)** one-way ANOVA with Dunnett´s correction and for **(B, F, H, I, M)** Kruskal Wallis test with Dunn´s multiple comparison test was used. * p < 0.05, ** p < 0.01, *** p < 0.001, and **** p < 0.0001 indicate significant differences between aldesleukin-treated or activated and vehicle-treated samples. CRP, c-reactive protein; GM-CSF, granulocyte-macrophage colony-stimulating factor; HLA, human leukocyte antigen; TRAIL-R, TNF-related apoptosis-inducing ligand receptor.

HepaRG surface marker expression was analysed fowllowing direct co-culture with CD8^+^ T cells (**Figure 4A**). In direct CD8^+^ T cell/HepaRG co-cultures stimulated. In direct CD8^+^ T cell/HepaRG co-cultures stimulated with high aldesleukin concentrations, HepaRG cells upregulated the surface expression of the cytotoxicity receptor Fas and concomitantly downregulated CD40, a molecule described to promote Fas-mediated hepatocyte apoptosis (57) (**Figures 4B, C**). Additionally, TRAIL-R1 was downregulated (**Figure 4D**). Upon ligand binding, TRAIL-Rs translocate to the nucleus (58), thus measuring decreased TRAIL-R1 surface expression might indicate receptor internalization. TRAIL-R2 was not altered by aldesleukin (**Supplementary Figure 8A**). Human hepatocytes show toxicity in response to TRAIL and express at least TRAIL-R1, -R2 and the decoy receptor 2 (59). In our assays, we only tested for TRAIL-R1 and -R2, as they are described to mediate apoptosis in normal (non-cancer) human hepatocytes (60). Peptides are presented to CD8^+^ T cells via HLA-B8 which corresponds to MHC Class I. The expression of HLA-B8 was diminished on HepaRG cells upon co-culturing with aldesleukin (≥ 100 IU/mL) treated CD8^+^ T cells or upon co-culture with CD3/CD28 and PHA-L activated CD8^+^ T cells (**Figure 4E**). HLA-DR was unchanged (**Supplementary Figure 8B**). The surface marker expression profile of co-cultures treated with high aldesleukin concentrations was similar to that in activation controls (**Figures 4B–E**).

Analyses of inflammatory markers released into the cell culture supernatants overall showed high variations between the tested donors (**Figures 4F–I**). In the direct CD8^+^ T cell/HepaRG co-culture, concentrations of the cytolytic molecule perforin were increased 8.4-fold by 5000 IU/mL aldesleukin compared to the vehicle control, but the increase did not reach statistical significance (**Figure 4F**). Also, a non-significant tendency towards an increase in granzyme B (6.8-fold, **Figure 4G**) was observed. This hints towards an apoptosis-inducing effect of aldesleukin-stimulated CD8^+^ T cells towards HepaRG cells, though inter-donor variations were high. IL-6 presents as a crucial pro-inflammatory cytokine for the induction of the hepatic acute phase response (61), and was increased upon aldesleukin stimulation (**Figure 4H**). Release of C-reactive protein (CRP) was induced by 5000 IU/mL aldesleukin, supporting a pro-inflammatory response in co-cultures stimulated with high aldesleukin concentrations (**Figure 4I**).

In total, the measured concentrations of the inflammatory markers released into the supernatant in the direct CD8^+^ T cell/HepaRG co-culture stimulated with aldesleukin were lower compared to those in response to the activation controls, despite CD8^+^ T cell viability was tendentially higher. In total, the measured concentrations of the inflammatory markers released into the supernatant in the direct CD8^+^ T cell/HepaRG co-culture stimulated with aldesleukin were lower compared to those in response to the activation controls (**Figures 4F–I**, **Supplementary Figures 8C–K**). Notably, in the transwell CD8^+^ T cell/HepaRG co-culture, aldesleukin stimulation of CD8^+^ T cells had no effect on HepaRG cell surface marker expression (**Supplementary Figures 9A-F**) inflammatory mediators (**Supplementary Figures 9G-P**), but increased CD8+ T cell viability (**Supplementary Figures 9Q**).

### 3.6 NK cell/HepaRG interactions influence hepatic surface marker expression and perforin release under high aldesleukin concentrations

To evaluate aldesleukin effects on hepatic surface marker expression and inflammatory molecule release in direct NK cell/HepaRG co-cultures, NK cells were pre-stimulated with aldesleukin for 44.5 h before addition to HepaRG cell layers for 3.5 h. On HepaRG cells, Fas surface expression was upregulated while TRAIL-R1 was downregulated (**Figures 4J, K**). For both markers, high aldesleukin concentrations elicited a comparable response to the activation control. HLA-DR was increased at ≥ 1000 IU/mL aldesleukin (**Figure 4L**). Surface expression of TRAIL-R2, CD40 and HLA-B8 was not influenced (**Supplementary Figures 10A-C**). Perforin release was significantly increased (**Figure 4M**) while granzyme B expression varied markedly between samples, obscuring any impact of aldesleukin (**Supplementary Figure 10D**). Also, changes in other relevant markers such as GM-CSF, IL-6 and CRP were donor-dependent and therefore, no impact of aldesleukin was observed with exception for NK cell viability, which was increased (**Supplementary Figures 10E-O**).

To characterize indirect NK cell/HepaRG interactions, primary human NK cells were stimulated and added to the upper chamber of transwell inserts with a HepaRG cell layer in the lower chamber. After 48 h, HepaRG surface markers and supernatants from the lower chamber were analysed. Aldesleukin-stimulated NK cells had no effect in indirect co-cultures on HepaRG surface protein expression (**Supplementary Figures 11A-F**) or pro-inflammatory mediator release (**Supplementary Figures 11G-R**). Aldesleukin increased NK cell viability in indirect co-cultures with HepaRG cells (**Supplementary Figures**). This suggests that direct cell-cell contact is likely required for aldesleukin effects. This is also represented by heatmap analyses of NK/HepaRG co-culture readouts, where overall in the direct but not the indirect co-culture effects, such as pro-inflammatory mediator release, was observed (**Supplementary Figure 12**).

In addition, in the CD8^+^ T cell/HepaRG and NK cell/HepaRG co-cultures, the function of the MRP2 transporter and the bilirubin glucuronidation capacity of hepatocytes were addressed to provide insight into possible bilirubin metabolism issues regarding conjugation and transport of this molecule which can lead to hyperbilirubinemia, a marker of aldesleukin-mediated hepatotoxicity (13). HepaRG cells were chosen to address hepatocyte function, as they retain physiologic PHH function including phase I and II enzyme activity (62). In detail, HepaRG cells express the enzyme UGT1A1 (63) which glucuronidates bilirubin (64) as well as a functional MRP2 transporter (65). Aldesleukin had no effects on MRP2 function or bilirubin glucuronidation (**Supplementary Figures 13, 14**) in any of the tested models.

### 3.7 Validation of the *in vitro* model with primary human hepatocytes

To mimic the human situation in liver cell culture models, PHHs are thought to be the most meaningful cell type (66). Based on the results described above, direct CD8^+^ T cell/HepaRG co-cultures were evaluated as the most pertinent for aldesleukin-mediated hepatic adverse events. We aimed to validate the results of this model with 5 d direct CD8^+^ T cell/PHH co-culture experiments for cytotoxicity assessment and 3 d pre-incubation of CD8^+^ T cells plus 2 days of co-culture for the analysis of surface markers and cytokines.

**Figure 5:**
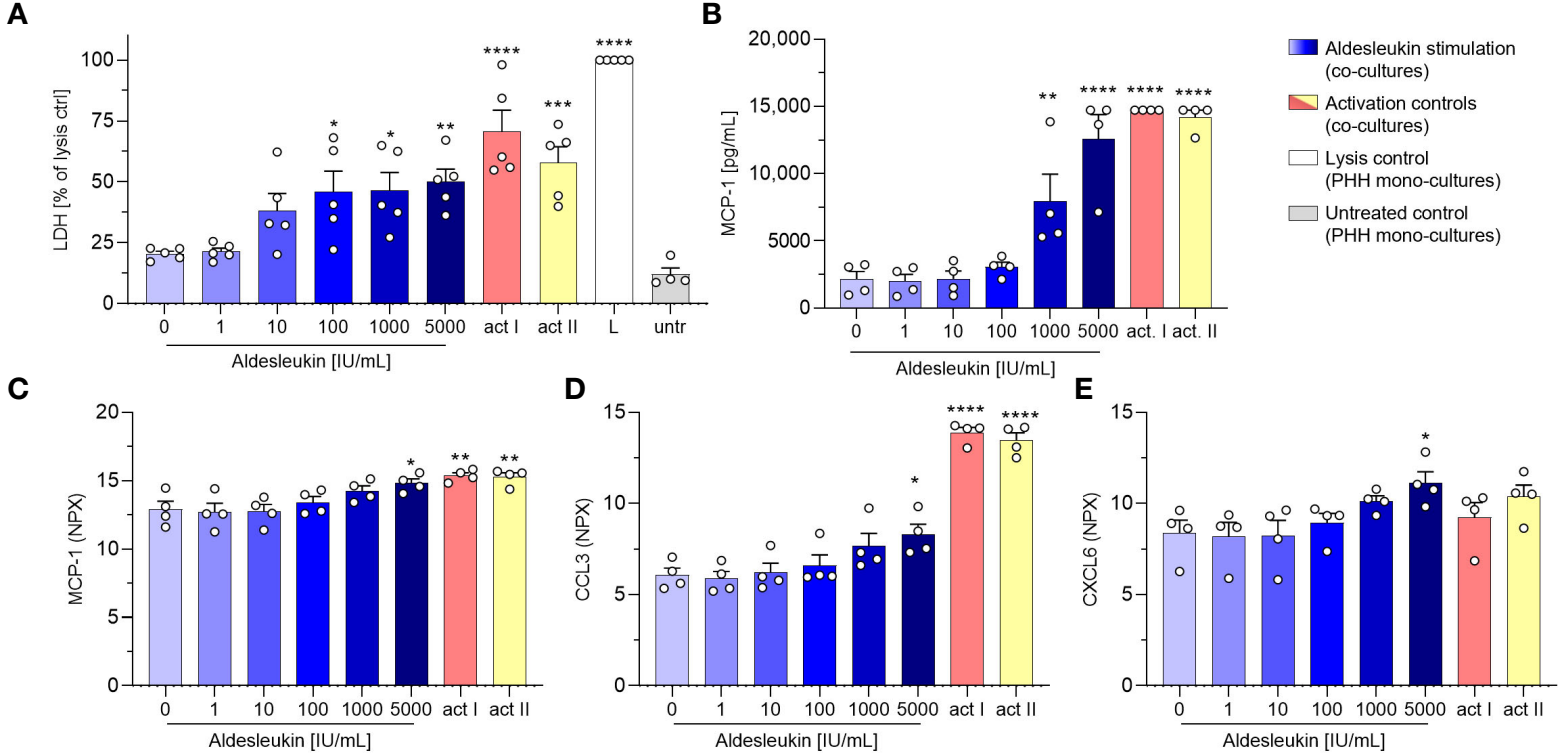
Aldesleukin induces cytotoxicity and pro-inflammatory mediator release in CD8^+^ T cell/primary human hepatocyte co-cultures. Primary human CD8^+^ T cells were stimulated with aldesleukin or activation controls (act I, CD3/CD28 + rhIL-2 activation; act II, PHA-L+ rhIL-2 activation) and co-incubated with primary human hepatocytes (PHHs). **(A)** For cytotoxicity assessment, CD8^+^ T cells were co-cultured with hepatocytes for 5 d. Lactate dehydrogenase (LDH) activity was determined via colorimetric assay and samples were referred to the lysis control. **(B-E)** For quantification of released proteins, 3 days pre-stimulated CD8^+^ T cells were added onto hepatocytes for 2 days. Proteins were quantified via **(B)** flow cytometry or via **(C-E)** proximity extension assay. Data are shown as mean ± SEM. *N* = 4 **(B-E)** and *N* = 5 **(A)** biological replicates were evaluated. Per measurement, one technical replicate was acquired. For statistical analysis, one-way ANOVA with Dunnett´s multiple comparison test was used. * p < 0.05, ** p < 0.01, *** p < 0.001 and **** p < 0.0001 indicate significant differences between aldesleukin-treated or activated and vehicle-treated samples. CCL3, Chemokine (C-C motif) ligand 3; CXCL6, Chemokine (C-X-C motif) ligand 6; L, Triton-X-lysed PHHs; MCP-1, monocyte chemoattractant protein 1; NPX, Normalized Protein eXpression; untr., untreated PHH monocultures.

Cytotoxicity was already detected at intermediate aldesleukin concentrations (≥ 100 IU/mL) and overall was comparable to co-cultures with HepaRGs. Notably, no change in LDH levels was seen when co-cultures of vehicle stimulated CD8^+^ T cells with PHHs were compared with supernatants from PHH mono-cultures (**Figure 5A**). Moreover, aldesleukin had no inherent cytotoxic effects on PHHs (**Supplementary Figure 7B**). Surface marker expression was not altered in PHH co-cultures and acquisition was possibly hindered by the sheer stress PHHs were exposed due during flow cytometry measurement (**Supplementary Figures 15A-F**). For some released molecules, a similar trend to that in CD8^+^ T cell/HepaRG co-cultures was observed (granzyme B, perforin, CRP), but inter-donor variation was pronounced and compromised outcomes (**Supplementary Figures 15G-Q**). Interestingly, Monocyte chemoattractant protein-1 (MCP-1/CCL2) was upregulated at higher aldesleukin concentrations and partially exceeded the upper limit of detection (**Figure 5B**).

Proteomic analyses with the Olink technique of the CD8^+^ T cell/PHH co-culture supernatants were aimed at gaining broader understanding of the impact aldesleukin stimulation has on protein release. Supernatants from four unique CD8^+^ T cell/PHH combinations were tested for the release of 92 immune-related proteins. The heatmap presentation of the averaged expression values shows that a variety of pro-inflammatory cytokines and chemokines were generated in response to aldesleukin and activation controls (**Supplementary Figure 16A**). 5000 IU/mL aldesleukin induced significant increases in pro-inflammatory mediators MCP-1, CCL3 and CXCL6 (**Figures 5C–E)**. Generally, inter-donor variations in aldesleukin-treated samples were considerably high. Donor-to-donor differences tended to be lower in activation control-treated samples in which, as expected, induction of proteins was more pronounced than in aldesleukin-treated samples. Differences in the quality of promoted response and mechanical differences between activation controls and aldesleukin are also reflected by principal component analysis (**Supplementary Figure 16B-D**).

### 3.8 Refinement of the *in vitro* model via implication of MdMs

**Figure 6:**
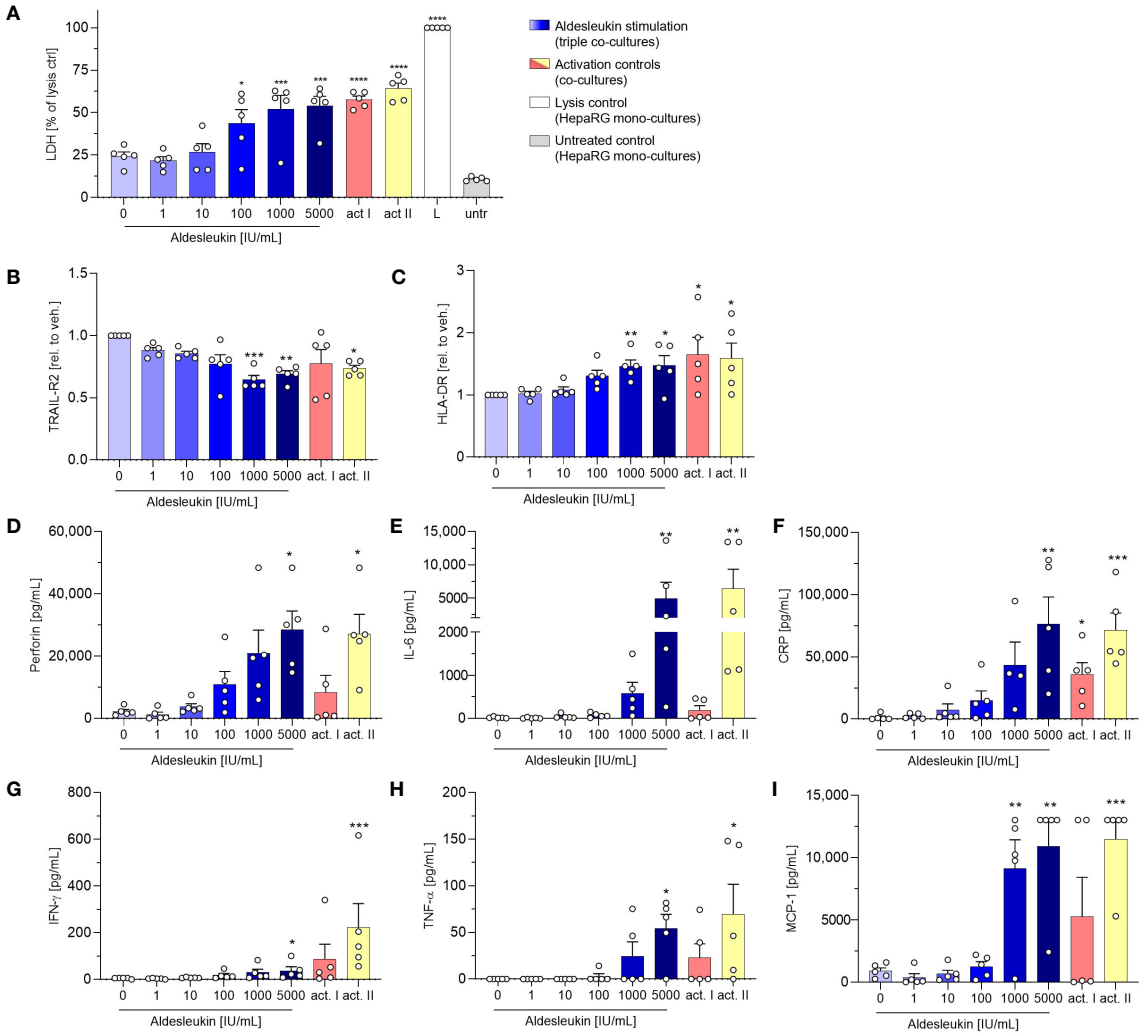
Addition of monocyte-derived macrophages to CD8^+^ T cell/HepaRG co-cultures enhances aldesleukin-induced pro-inflammatory effects. Primary human CD8^+^ T cell/monocyte-derived macrophage (MdM) co-cultures were treated with aldesleukin or activation controls (act I, CD3/CD28 + rhIL-2 activation; act II, PHA-L+ rhIL-2 activation) and co-cultured with HepaRG cells in direct co-cultures. **(A)** For cytotoxicity assessment, CD8^+^ T cell/MdM mixtures were co-cultured with HepaRG cells for 5 d. Colorimetric assessment of lactate dehydrogenase (LDH) activity was conducted to measure cell-mediated cytotoxicity. Samples were referred to the lysis control. **(B-I)** 3 days pre-stimulated CD8^+^ T cell/MdM co-cultures were co-cultured with HepaRG cells for additional 2 days. **(B, C)** Hepatic surface marker expression was measured with flow cytometry and the mean fluorescence intensity was related to the vehicle control. **(D-I)** Pro-inflammatory mediators were quantified via flow cytometry; CRP was determined via ELISA. Data are shown as mean ± SEM. *N* = 5 biological replicates were evaluated. Per biological replicate, one technical replicate was acquired. For statistical analysis of **(I)** one-way ANOVA with Dunnett´s multiple comparison test was used and for analysis of **(A-I)** Kruskal Wallis test with Dunn´s correction was applied. * p < 0.05, ** p < 0.01, *** p < 0.001 and **** p < 0.0001 indicate significant differences between aldesleukin-treated or activated and vehicle-treated samples. CRP, c-reactive protein; IFN-γ, Interferon-γ; IL-6, Interleukin-6; L, Triton-X-lysed HepaRG control; MCP-1, monocyte chemoattractant protein 1; untr., untreated HepaRG monocultures.

In view of the upregulation of potentially macrophage-activating proteins in CD8^+^ T cell/HepaRG co-cultures and since macrophage activation has been implicated in aldesleukin-mediated hepatotoxicity previously (19), we refined the direct CD8^+^ T cell/HepaRG co-culture model by adding MdMs, creating a more complex, direct CD8^+^ T cell/MdM/HepaRG co-culture model, termed triple co-culture. In terms of cytotoxicity, in triple co-cultures, aldesleukin concentrations ≥ 100 IU/mL dose-dependently increased LDH release to a similar degree as in the preceding direct co-culture experiments (**Figure 6A**). Analysis of surface marker expression on HepaRG cells, interestingly, only showed down-regulation of TRAIL-R2 expression and upregulation of HLA-DR starting at aldesleukin concentrations ≥ 100 IU/mL while the other surface markers were unchanged (**Figures 6B, C** and **Supplementary Figure 17A-D**). Of all tested models, the overall highest protein levels were detected in triple co-cultures, indicating synergistic effects in this system. There was a distinct increase in perforin at ≥1000 IU/mL aldesleukin and IL-6 and CRP were also strongly increased at 5000 IU/mL aldesleukin. Additionally, IFN-γ was upregulated at the highest aldesleukin concentration and was increased in all but one donor, in which no TNF-α could be detected at all. Further, MCP-1 was distinctly increased at ≥1000 IU/mL aldesleukin and partially exceeding the detection limit (**Figures 6D–I**). There was a tendency of aldesleukin-induced granzyme B expression, however the inter-donor variation was considerably high (**Supplementary Figures 17E**) and the rest of the cytokines assessed was not influenced by aldesleukin stimulation (**Supplementary Figures 17F-J**). Comparing the activation controls, PHA-L was more potent in inducing protein release into the supernatant than CD3/CD28 activation, which was in strong contrast to the previous co-culture experiments. Taken together, the triple co-culture permits a novel approach to the potential mechanism of aldesleukin-induced hepatotoxicity. In terms of cytotoxicity, the three direct co-culture models (CD8^+^ T cell/HepaRG, CD8^+^ T cell/PHH and triple co-culture) are comparable.

### 3.9 Analysis of relevant markers from co-culture models with CD8^+^ T cells

**Figure 7:**
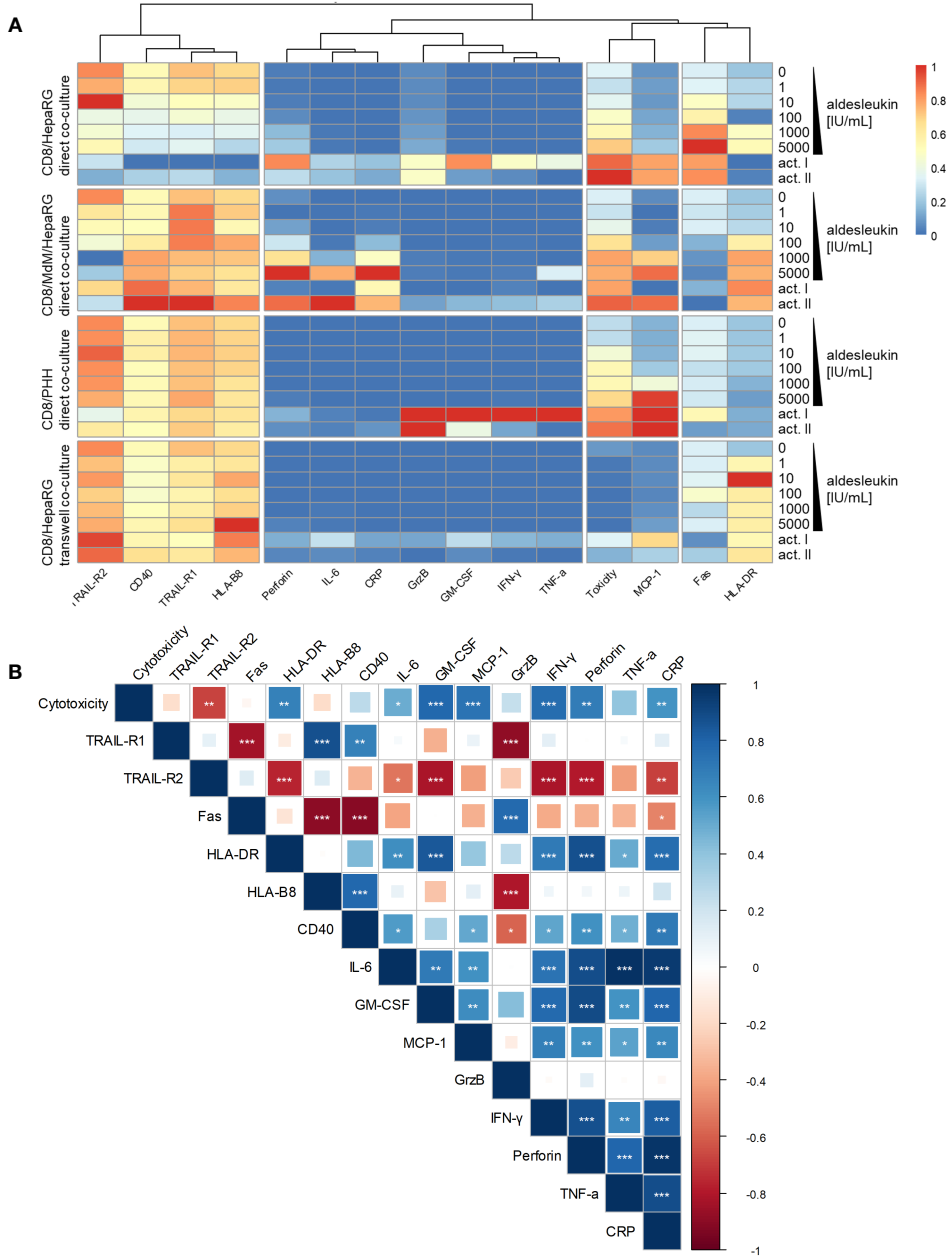
Heatmap and correlation analysis of CD8^+^ T cell implying co-culture results. **(A)** A heatmap was visualized representing regulation of cytotoxicity, hepatic surface marker and cytokine results in all co-culture models that have been tested with CD8^+^ T cells. The columns are clustered so that similarly regulated markers are ordered next to each other. **(B)** Correlation analysis of cytotoxicity, hepatic surface marker and cytokine regulation in aldesleukin-stimulated direct co-cultures implying CD8^+^ T cells was performed. * p < 0.05, ** p < 0.01 and *** p < 0.001 indicate significant correlations between the readouts.

Heatmap and clustering analysis of all co-culture models with CD8^+^ T cells indicates that MCP-1 and cytotoxicity are similarly regulated over all models (**Figure 7A**). Correlation analysis of results from aldesleukin-stimulated direct co-cultures with CD8^+^ T cells shows that TRAIL-R2 is negatively and HLA-DR, IL-6, GM-CSF, MCP-1, IFN-γ, perforin, and CRP are positively correlated with cytotoxicity (**Figure 7B**).

## 4 Discussion

Modelling immune-mediated drug-induced hepatotoxicity is challenging as many of the underlying mechanisms remain unclear and animal experiments provide limited predictive power (6). Many efforts have been made to develop immune-inflammatory, *in vitro* hepatotoxicity models by co-culturing immune cells with hepatocytes, for instance by addressing THP-1 cell line/HepG2, primary human NK cell/HepG2, or Kupffer cell/PHH co-cultures (6) or by co-culturing of PBMCs with HepaRG cells (67). Also *in vivo* models are central to current research. With regard to adverse reactions to antibiotics, a flucloxacillin DILI mouse model was assessed. A key finding of this model was that hepatotoxic effects produced by CD8^+^ T cells were restricted by the tolerogenic environment in the liver and the absence of T_reg_ cells may promote cytotoxic T cell responses through lack of IL-2 depletion (68). A humanized mouse model was used to evaluate IL-2-mediated toxicities in different organs. High dose IL-2 distinctly increased T cell numbers in the livers of the animals and the authors underlined the role of the effector T/Treg cell equilibrium in IL-2-mediated adverse events (35). This latter mouse model could be used to assess the hepatotoxic effects of high dose aldesleukin in detail.

To model immune-mediated hepatotoxicity elicited by the immunomodulatory drug aldesleukin, we developed several hepatotoxicity test systems starting with simple models consisting of primary immune cells and a hepatocyte cell line (CD8^+^ T cell/HepaRG, NK cell/HepaRG), increasing complexity by adding a further immune cell type (CD8^+^ T cell/MdM/HepaRG) or switching to PHHs (CD8^+^ T cell/PHH). Our results clearly demonstrate that CD8^+^ T cells are more relevant for the mediation of aldesleukin-mediated hepatotoxicity than NK cells, direct contact between immune cells and hepatocytes being required and the addition of macrophages enhancing aldesleukin-mediated effects. However, we observed comparable cytotoxicity effects and similar trends for protein release between the hepatocyte cell line and the PHHs, indicating that HepaRG cells are a suitable model for hepatotoxicity.

Taken together, to evaluate immune-mediated hepatotoxicity induced by biologicals, we have established that the following steps should be addressed: 1) identification of relevant immune cells, 2) co-culture/multi-culture of identified immune cells with hepatocyte-like cells, 3) evaluation of results with primary human cells.

In our transwell co-culture experiments, no hepatotoxic effects were observed and no pro-inflammatory mediators were induced by aldesleukin. In the context of T cell-mediated hypersensitivity to the antibiotic dapsone, an indirect co-culture model consisting of T cells and PHHs was developed. This system was shown to be effective for the evaluation of the influence of *in situ* generated metabolites on T cell activation, without direct contact between hepatocytes and immune cells (69). Moreover, hepatic exosomes might be implicated in immune cell activation by small molecules. In exosomes, drug-modified protein fragments are delivered to dendritic cells which present these peptides to T cells via HLA (70). To our knowledge, no similar studies were conducted with biologics. Addition of antigen presenting cells to our CD8^+^ T/HepaRG cell model might provide useful insights if a similar exosome-mediated mechanisms is induced.

In direct NK cell/HepaRG co-cultures, death receptors Fas and TRAIL-R1 were regulated on HepaRG cells in an aldesleukin- dependent manner and perforin was increased. Binding of TRAIL, which was increased on mono-cultured NK cells upon aldesleukin stimulation, to its receptors TRAIL-R1 and -R2, confers apoptosis (71). However, NK cell/HepaRG co-cultures only moderately captured aldesleukin-mediated cytotoxic effects. No increase in CRP was observed, possibly because the co-culture time of 3.5 h might be too short to induce CRP release which, in humans, starts 2 h after induction of the acute phase response (72). Such a short co-culture time was necessary as basal cytotoxicity of NK cells towards the HepaRG cell line was high. Therefore, we established a protocol for measuring NK cell cytolytic activity towards HepaRG cells. In this assay, a small but significant increase in cytolysis was seen in NK cell/HepaRG co-cultures stimulated with 5000 IU/mL aldesleukin or the IL-15/IL-21 activation control. The effects observed were presumably influenced by the NK cell expansion protocol which yielded activated NK cells. We chose this protocol not only to expand NK cells, but also as it upregulated CD56, a marker for liver-resident NK cells (73). It would therefore be favourable to address NK cell cytotoxicity towards hepatocytes in a model in with liver-derived NK cells in order to mimic the situation in this organ more closely. Indirect NK cell/HepaRG co-cultures, despite extending the incubation time compared to the direct co-culture assays, were not a suitable model to capture the effects of activated NK cells on hepatocytes as neither an impact on surface marker expression nor on cytokine release could be detected. A possible explanation for the lack of effects in indirect co-cultures would be that NK-target cell contact and the formation of a immunological synapse facilitates NK cell cytotoxicity (74).

Direct CD8^+^ T cell/HepaRG co-culture was the most promising model to mimic aldesleukin-mediated hepatotoxicity, as a concentration-dependent increase in LDH was seen, in agreement with increases in hepatic aminotransferase observed in patients upon high dose aldesleukin treatment (13). In the CD8^+^ T cell/PHH and CD8^+^ T cell/MdM/HepaRG co-cultures, we observed similar toxicities as in the CD8^+^ T cell/HepaRG co-cultures. However, cytotoxicity was already induced at an intermediate aldesleukin concentration (≥ 100 IU/mL), indicating slightly increased sensitivity of these models. Indirect co-cultures did not lead to measurable cytotoxic effects of CD8^+^ T towards HepaRG cells, indicating the importance of direct cell-cell contact. The inter-donor variability was high, which mirrors the situation in the clinic, since upon aldesleukin therapy, increases in liver enzyme levels are common, but severe increases only occur in a fraction of patients (14). High variabilities between donors are also reviewed in the literature (6), indicating that to evaluate immune-competent hepatotoxicity models, larger populations of donors should be characterised.

We aimed to assess the non-specific activation of immune cells to overcome the tolerogenic environment of the liver in co-culture experiments that implied immune cells and hepatocytes from different donors. In our models, there was no significant difference in LDH release between monocultured hepatocytes (PHHs or HepaRG cells) and vehicle stimulated co-cultures with immune cells, indicating that in these allogeneic systems, cytotoxicity exerted by alloreactive T cells played a secondary role. Aligning with our observations, in similar *in vitro* immune cell/hepatocyte co-culture models it was reported that alloreactivity plays a minor role (67, 75). This is illustrated by the fact that in human liver transplantation, for the survival of liver grafts, histocompatibility between donor and recipient is not a must and in certain cases, complete withdrawal of immunosuppressive therapy is possible (76). In a transgenic mouse model, hepatocytes activated alloreactive CD8^+^ T cells, but the activated T cells prematurely underwent apoptosis due to the lack of survival signals. Thus, hepatocytes promoted liver tolerance instead of creating a immunological memory (77). A similar situation might arise in the vehicle-stimulated co-culture conditions. There, the tolerogenic environment is presumably maintained but the addition of high aldesleukin concentrations overcomes this tolerance effect. The use of such allogenic models may not be ideal to model the processes in cancer patients who did not receive liver transplantation. However, donor-matched cells were not accessible. Future experiments with donor-matched models and pulsing of hepatocytes with foreign antigens would mimic the situation in patients more closely.

By death receptor-ligand engagement, the extrinsic pathway of apoptosis is initiated (78). Therefore, the regulation of the death receptors Fas and TRAIL-R1 on HepaRG cells in direct CD8^+^ T cell/HepaRG co-cultures points towards the induction of extrinsic apoptosis in HepaRG cells. Furthermore, CD40 activation can lead to increased hepatocyte Fas ligand expression and enhance hepatocyte death in an autocrine or paracrine manner by crosslinking with Fas (57). Simultaneously, there was a tendency towards perforin and granzyme B increase pointing towards the induction of a second apoptosis pathway, mitochondrial apoptosis, as it was shown that granzyme B, entering the cell by aid of perforin, induces this apoptosis pathway in the hepatocytes (79).

CRP is an important protein of the acute phase response and is mainly produced in hepatocytes in response to pro-inflammatory cytokines, notably IL-6, but also TNF-α. CRP can promote pro-inflammatory processes by induction of cytokines, such as MCP-1 (80). These effects were most pronounced in direct CD8^+^ T cell/MdM/HepaRG co-cultures.

PHHs were employed to validate the results obtained in the direct CD8^+^ T cell/HepaRG co-cultures. Similar tendencies in cytotoxic molecule and cytokine release were found, though high donor to donor variability arose in aldesleukin-treated samples. MCP-1 was significantly increased by high aldesleukin concentrations in samples analysed by flow cytometry and Olink technology. Proteomic analysis with Olink technology additionally revealed an increase in MIP-1α/CCL3 and CXCL6. The chemokine MIP-1α can be secreted by mature hematopoietic cells, but also by hepatocytes and promotes the recruitment of leukocytes to the site of inflammation (81, 82). CXCL6 is known as a neutrophil activating chemokine (83). In fibrosis patients, increases in CXCL6 levels are present in sera and liver tissue, rendering CXCL6 a possible marker for liver fibrosis. *In vitro*, CXCL6 promotes the secretion of TGF-β by Kupffer cells and conditioned medium derived from CXCL6-stimulated Kupffer cells activates hepatic stellate cells (84). The authors of this study, however, did not assess the expression of other chemokines by Kupffer cells. From these data, we assume that CXCL6 and CCL3 might contribute to macrophage activation which, in turn, could enhance the aldesleukin response in our model. Other analytes were modified in a donor dependent manner, which precludes discussion of all these molecules, underlining that preferably larger donor pools have to be evaluated to obtain meaningful results.

**Figure 8:**
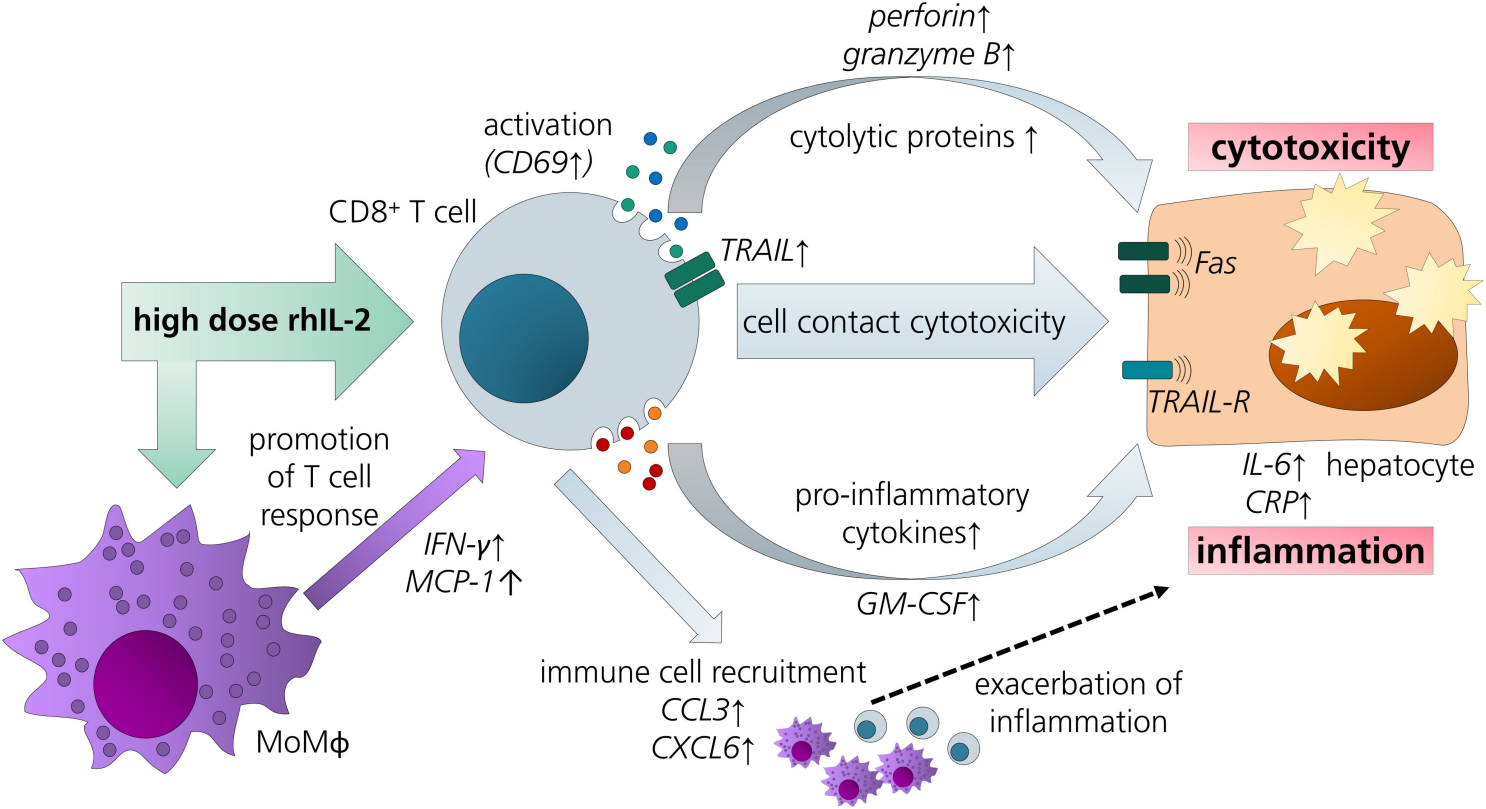
Overview of potential pathways and key players in aldesleukin-mediated hepatotoxicity. Upon high-dose aldesleukin (rhIL-2), CD8^+^ T cells were activated and in direct co-cultures with the HepaRG cell line and primary human hepatocytes, cytotoxic molecules such as perforin and pro-inflammatory mediators such as c-reactive protein were induced. Direct cell-cell contact was necessary to induce a cytotoxic effect. Proteomic analyses point towards the recruitment of further immune cells, such as T cells and monocytes. Addition of monocyte-derived macrophages to the co-cultures enhanced the pro-inflammatory response. CCL3, Chemokine (C-C motif) ligand 3; CXCL6, Chemokine (C-X-C motif) ligand 6; CRP, C-reactive protein; GM-CSF, granulocyte-macrophage colony-stimulating factor; IL-6, interleukin 6; IFN-γ, interferon gamma; MCP-1, monocyte chemoattractant protein 1; MoMΦ, monocyte-derived macrophage; TRAIL, TNF-related apoptosis-inducing ligand; TRAIL-R, TNF-related apoptosis-inducing ligand receptor.

The results discussed above led us to refine the model by addition of MdMs - as a surrogate for hepatic macrophages - to CD8^+^ T cell/HepaRG co-culture (triple co-culture). Indeed, animal studies also suggest a contribution of macrophages to IL-2-mediated hepatotoxicity (19). In our triple co-culture, a similar aldesleukin-induced cytotoxic effect was observed as in the CD8^+^ T cell/PHH co-culture. In the triple co-cultures, there was a more pronounced induction of perforin, MCP-1 and IFN-γ. Also, in terms of acute phase response, IL-6 and CRP were more markedly induced than in the co-cultures employing only two cell types. This observation might be related to the increased TNF-α release by high aldesleukin concentrations in triple co-cultures. TNF-α was proposed as a key molecule for aldesleukin-mediated hepatotoxicity, as its transcription was augmented in the livers of mice treated with high concentrations of aldesleukin and in turn might lead to activation of further immune cells as well as liver endothelial cells (19). Also, for other organ toxicities induced by aldesleukin, TNF-α is thought to play a crucial role (13), besides IFN-γ (85), which was also increased in triple co-cultures. In many cases, the highest aldesleukin concentration tested produced effects to a comparable degree as the PHA-L activation control, which was not the case for CD8^+^ T cell/HepaRG co-cultures in which the aldesleukin response was lower. Taken together, these data point towards cooperative effects between the innate and the adaptive arms of immunity in the induction of aldesleukin-mediated hepatotoxicity (**Figure 8**).

The correlation analysis of the results for aldesleukin-treated direct CD8^+^ T cell/HepaRG, CD8^+^ T cell/PHH and CD8^+^ T cell/MdM/HepaRG co-cultures allows specific assessment of aldesleukin-mediated effects. Here, cytotoxicity as central readout of all the assays was positively correlated with a variety of cytotoxic and pro-inflammatory molecules that are also reflected by clinical data with cancer immunotherapy: IL-6 and CRP are increased in liver cancer patients developing immune-related adverse events following combination immunotherapy with PD-1 checkpoint inhibitors (86). In patients with checkpoint inhibitor mediated hepatitis, increased frequencies of perforin expressing peripheral CD8^+^ T cells were detected, additionally increases in granzyme B were measured which was not correlated in our analyses. Also, *ex vivo* LPS-activated macrophages from hepatitis patients secreted higher levels of pro-inflammatory mediators (i.a. IFN-γ, TNF-α, IL-6) compared to macrophages from healthy volunteers. Liver histology points towards co-localization of CD8^+^ T cell/macrophage aggregates (33), which fits to our hypothesis of cooperative effects between these two cell types. To gain an in-depth understanding of the molecular mechanisms of aldesleukin-induced immune-mediated hepatotoxicity, a more complex model, preferably with donor-matched immune cells and liver-resident cell types, would be favourable to generate a human-relevant, immunocompetent hepatotoxicity test system.

Nguyen and colleagues addressed the effect of pro-inflammatory cytokines on pro-inflammatory mediator release, metabolizing enzymes (such as CYP3A4) and drug transporters in co-cultures of Kupffer cells and hepatocytes (87). IL-6 induced CRP mRNA as well as cytokine release (IL-8, TNF-α and IFN-γ). IL-6 also downregulated the function and expression of several metabolizing enzymes and efflux transporters, but not of MRP2. In contrast, IL-2 did not lead to the production of these cytokines, only moderately decreased CYP3A4 activity and the authors attributed this to the lack of IL-2Rβ mRNA expression (87). Therefore, Kupffer cells alone might not be sufficient to induce IL-2 mediated effects. The results obtained with IL-6 support our hypothesis, that the cross-talk from multiple immune cell types may be necessary to induce aldesleukin-mediated effects.

One hallmark of aldesleukin-mediated hepatotoxicity is hyperbilirubinemia (13). One of the limitations of the study was the failure to detect alterations in bilirubin metabolism. We tested hepatocyte function after 2 d (CD8^+^ T cells) or 3.5 h (NK cells) of direct co-culture and possibly, elongated co-culture times would permit detection of effects on MRP2 transporter function or bilirubin glucuronidation. But due to the increasing cytotoxicity with time, it would not be possible to discriminate whether the effect on the bilirubin metabolism is due to cytotoxicity or to a functional loss of bilirubin metabolizing enzymes. Alternatively, in our simple models, crucial mediators for impaired bilirubin metabolism might be missing. This leads to another limitation of the study: the reduced complexity hinders a comprehensive understanding of the situation in humans. However, the successive development of models with increasing complexity, as shown in the present work, will facilitate the precise identification of possible key players and mechanisms in drug-induced immune-mediated hepatotoxicity.

Validation of the results via more sophisticated models with preferably primary human (liver) cell types would provide a clearer picture of the processes involved in aldesleukin-induced immune-mediated hepatotoxicity. Such advanced models include PHH spheroid/Kupffer cell co-cultures which were already used to recapitulate toxicity responses to trovafloxacin and acetaminophen (88). Also cancer organoids have already been successfully co-cultured with PBMCs to assess the efficiency of T cell-induced tumour killing (89), and a similar setup might be applied to assess T cell-mediated hepatotoxicity. Moreover, 3D microphysiological liver sinusoids mimic key liver functions and can be transfused with immune cells (90). As in all models comprising primary human cells, attention has to be paid to inter-donor variability (91) which might necessitate pre-screening of suitable donors or the inclusion of multiple donors per experiment, which was done in this study.

We focused on the development of immune-mediated hepatotoxicity models that can be translated to other biologicals, thus we targeted “general” immune-toxicity mechanisms, such as the pro-inflammatory action of aldesleukin. However, genetic predispositions might favour aldesleukin-mediated adverse events. For instance, HLA-risk alleles have been described to play a role in DILI (92). Moreover, drug reactions with eosinophilia and systemic symptoms (DRESS) syndrome, which often involve the liver, can be induced by immunomodulatory therapies such as the monoclonal antibodies (93). DRESS is suggested to be linked to specific HLA-subtypes, hapten presentation, and antiviral responses, all mechanisms that are linked to increased T cell immune activation (94). Consequently, we cannot rule out that aldesleukin may induce similar side-effects through protein-hapten or other interactions with the TCR.

To translate these findings to other immunomodulatory biologicals that induce immune-mediated DILI, such as immune checkpoint inhibitors (95) experiments should be adapted, for instance in terms of choice of cell types used in the models and outcomes should be compared to the clinical manifestation of these adverse reactions. This will aid the development of meaningful *in vitro* models that may be used in the future to predict hepatotoxic effects of novel immunomodulatory therapies.

## 5 Conclusion

There is a need for meaningful pre-clinical *in vitro* models to assess drug-induced immune-mediated hepatotoxicity. In the present study, we developed test models with aldesleukin as a model biological. We showed that CD8^+^ T cells may be relevant cell types for aldesleukin-induced hepatic adverse events by inducing cytotoxicity and the release of pro-inflammatory mediators which in turn contribute to the recruitment of further immune cells, such as macrophages that exacerbate inflammation. A correlation study supported these findings, as hepatotoxicity and cytotoxic as well as pro-inflammatory mediators such as perforin, GM-CSF, MCP-1 and CRP were positively correlated. The models presented, as well as our approach to assay design, including the activation controls, offer a step forward in the development of more comprehensive immuno-inflammatory *in vitro* models. In the future, similar models could be employed for the prediction of adverse events induced by novel compounds with a similar mode of action or other biologicals.

## Data availability statement

The raw data supporting the conclusions of this article will be made available by the authors, without undue reservation.

## Ethics statement

The studies involving humans were approved by Medical Faculty of Leipzig University (Ethical vote: registration number 322/17-ek, date 2020/06/10 ratified on 2021/11/30 and registration number 238/22-ek, date 2022/07/18). The studies were conducted in accordance with the local legislation and institutional requirements. The participants provided their written informed consent to participate in this study.

## Author contributions

LR: Conceptualization, Data curation, Investigation, Writing – original draft, Writing – review & editing. SL: Data curation, Investigation, Writing – original draft. NZ: Data curation, Writing – original draft. DT: Data curation, Writing – original draft. PW: Investigation, Writing – original draft. GD: Writing – original draft. AS: Writing – original draft. PH: Conceptualization, Writing – original draft. MP: Conceptualization, Funding acquisition, Writing – original draft. SS: Conceptualization, Data curation, Funding acquisition, Writing – original draft, Writing – review & editing.
