# Supplementary material for: Immuno-inflammatory in vitro hepatotoxicity models to assess side effects of biologicals exemplified by aldesleukin
Source: Front Immunol. 2023 Nov 17;14:1275368. doi: 10.3389/fimmu.2023.1275368 (PMC10693457; doi:10.3389/fimmu.2023.1275368)
Supplement: Supplementary file 1 [file Presentation_1.pdf]

Supplemental Figure 1

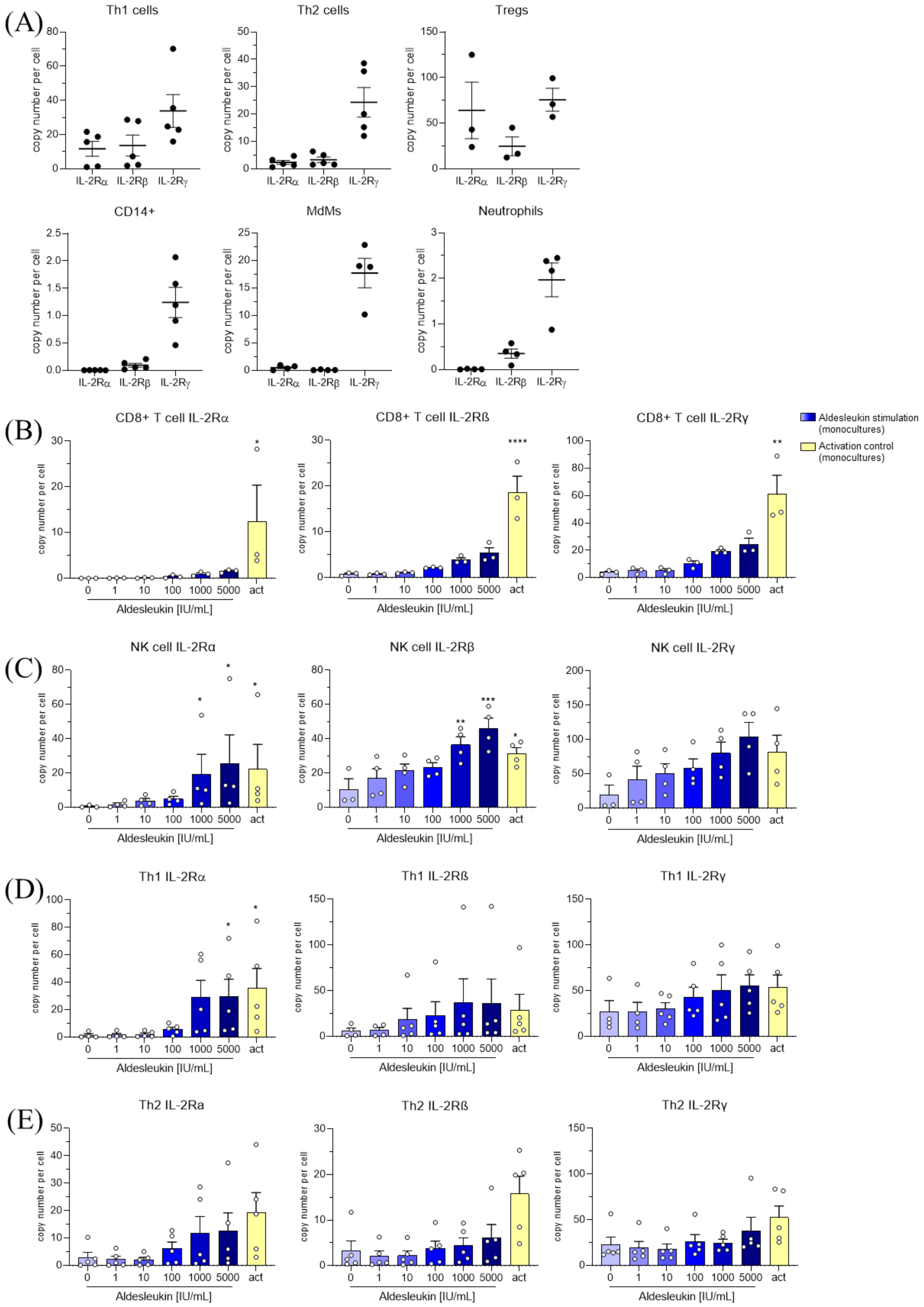

Supplemental Figure 1: IL-2R expression in primary human immune cells. Primary human immune cells were isolated from buffy coats. T helper (Th) 1 and Th2 cells were differentiated from naïve CD4+ T cells. Regulatory T cells (T_{regs}) were isolated as $CD4^{+}CD25^{+}$ cells and expanded for 14 days in the presence of CD3/CD28 MACSiBead Particles (Miltenyi Biotec, Bergisch Gladbach, Germany) and 500 IU/mL rhIL-2. $CD14^{+}$ T cells were isolated and polarized to monocyte-derived macrophages (MdMs) with 10 mg/mL GM-CSF for 7 days. Neutrophils were isolated via density gradient centrifugation. (A) Baseline IL-2R mRNA expression and (B-E) IL-2R mRNA expression upon stimulation with aldesleukin or activation control (PHA-L + rhIL-2 in B, D, E; CD2/CD335 + rhIL-2 in C) were determined via qPCR. Number of biological replicates: (A) $N = 5$ for Th1, Th2 and $CD14^{+}$ cells, $N = 4$ for MdMs and neutrophils, $N = 3$ for T_{regs}. For (B) $N = 3$, for (C) $N = 4$, for (D, E) $N = 5$. Technical duplicates were measured. Data are shown as mean ± SEM. For (A), no statistical analysis was performed. For (B-E), one-way ANOVA with Dunnett´s correction or Kruskal Wallis test with Dunn´s multiple comparison test was used. * $p < 0.05$, ** $p < 0.01$, *** $p < 0.001$ and **** $p < 0.0001$ indicate significant differences between aldesleukin-treated or activated and vehicle-treated samples.

Supplemental Figure 2

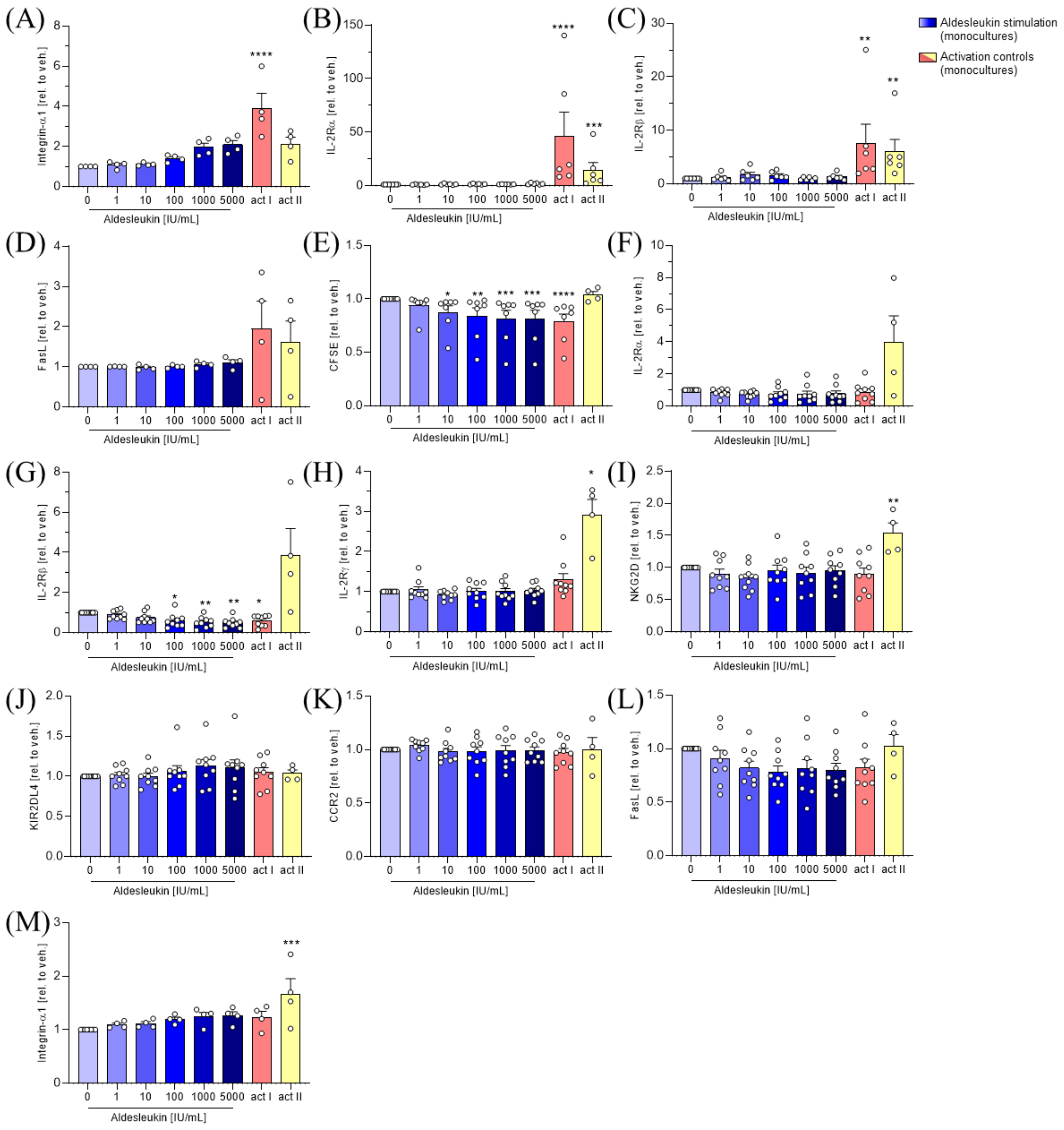

Supplemental Figure 2: Effect of aldesleukin on CD8$^+$ T and NK cell surface marker expression. (A-D) Primary human CD8$^+$ T cells were isolated from buffy coats and stimulated with aldesleukin or activation controls (act I, CD3/CD28 + rhIL-2 activation; act II, PHA-L+ rhIL-2 activation). (B-M) Primary human NK cells were isolated from buffy coats, expanded for 11-12 days and stimulated for 2 d with aldesleukin or activation controls (act I, CD2/CD335 + rhIL-2 activation; act II, CD2/CD335 + rhIL-15/21 activation). Surface marker expression was analysed with a MACSQuant® Analyzer 10 and the mean fluorescence intensity was related to the vehicle control. Data are shown as mean ± SEM. Number of biological replicates: $N = 4$ (A, D), $N = 6$ (B, C), $N = 7$ (E), $N = 9$ (F-L). In some cases, there are fewer biological replicates for the activation controls. One technical replicate was acquired per biological replicate. For statistical analysis of (A, D, I, K-M) one-way ANOVA with Dunnett´s correction, for analysis of (B, C, E- H, J) Kruskal Wallis test with Dunn´s multiple comparison test was used. * $p < 0.05$, ** $p < 0.01$, *** $p < 0.001$ and **** $p < 0.0001$ indicate significant differences between aldesleukin-treated or activated and vehicle-treated samples. Abb.: CCR2, C-C chemokine receptor type 2; CFSE, carboxyfluorescein succinimidyl ester; FasL, Fas ligand; IL-2R, interleukin-2 receptor; KIR2DL4, Killer Cell Immunoglobulin Like Receptor, Two Ig Domains And Long Cytoplasmic Tail 4; NKG2D, Natural Killer group 2D.

Supplemental Figure 3

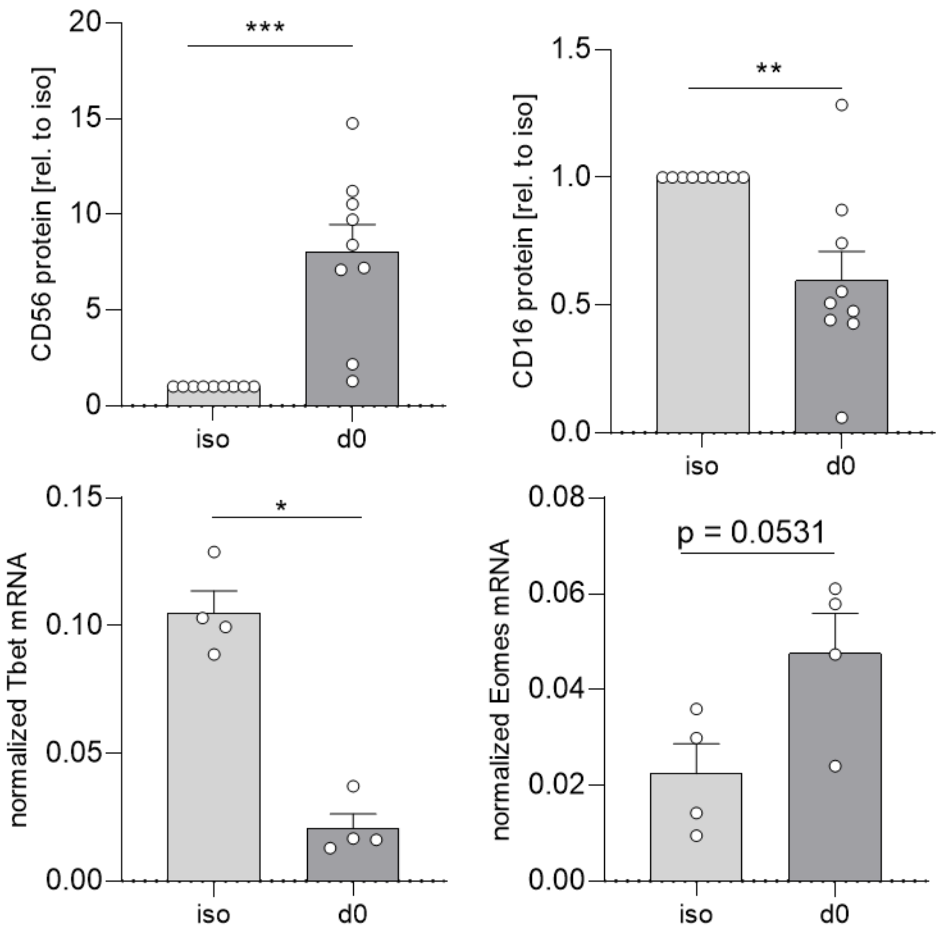

Supplemental Figure 3: Effect of NK cell expansion on NK cell marker expression. NK cells were isolated from buffy coats and expanded for 11-12 days with the human NK Cell Activation/Expansion Kit (Miltenyi Biotec, Bergisch Gladbach, Germany) and 500 IU/mL rhIL-2. Surface marker expression was determined via flow cytometry and mRNA expression was determined via qPCR and referred to the housekeeping genes βActin and TBP-1 *N* = 9 biological replicates for CD56 and CD16 acquisition, *N* = 4 for qPCR. Flow cytometry measurements were acquired with one technical replicate, for qPCR two technical replicates were measured. Data are shown as mean ± SEM. For statistical analysis, unpaired T test was used. * p<0.05, ** p<0.01 and *** p < 0.001 indicate significant difference between freshly isolated (iso) and d0 samples (after expansion). Abb.: Eomes, Eomesdermin; iso, day of isolation from buffy coat; TBP-1, TATA-box binding protein-1; T-bet, T-box transcription factor TBX21.

Supplemental Figure 4

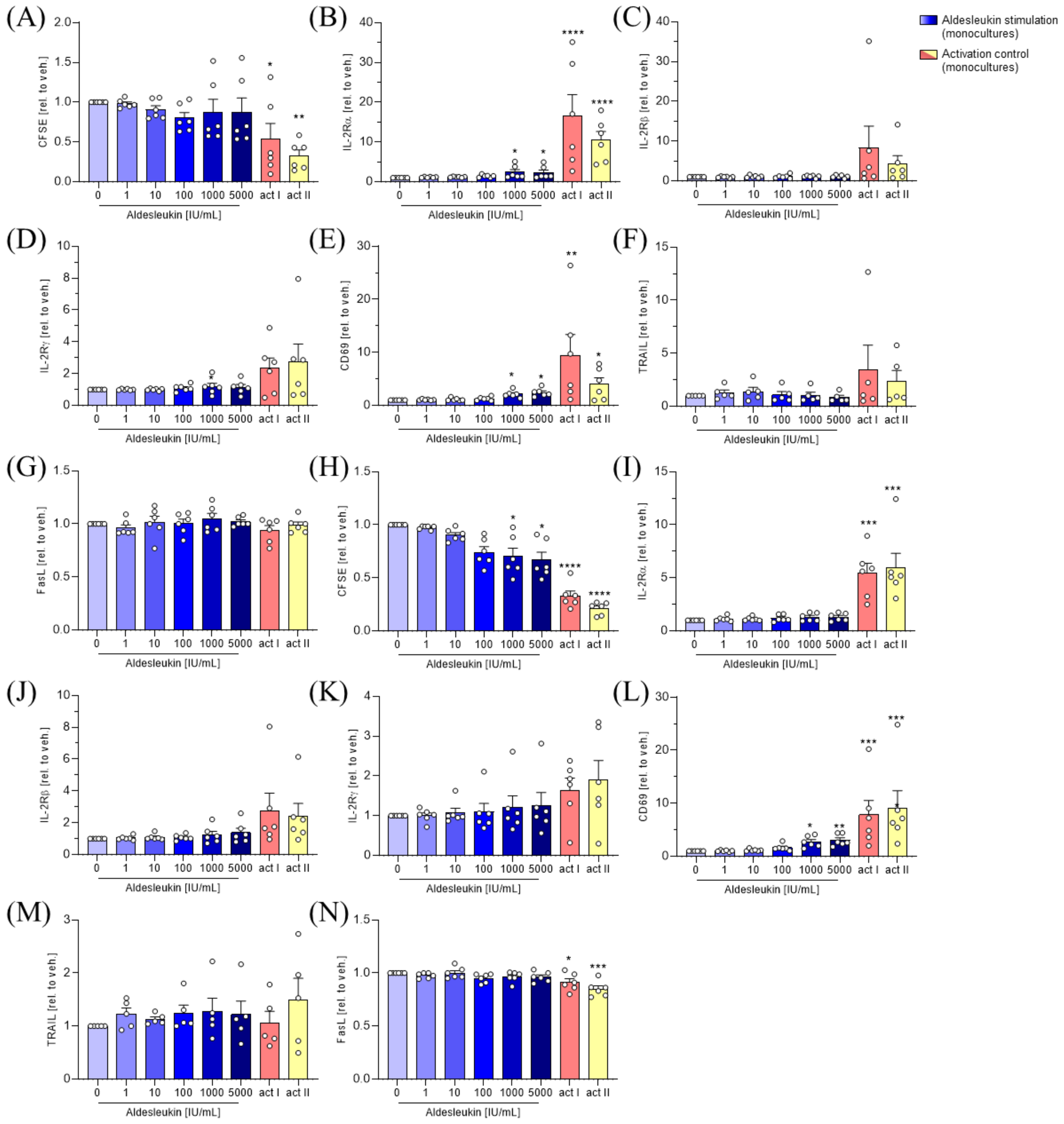

Supplemental Figure 4: Influence of aldesleukin on T helper cells. T helper (Th) 1 and Th2 cells were differentiated from primary human naïve $CD4^+$ T cells. (A-G) Th1 and (H-N) Th2 cells were stimulated for 5 days with aldesleukin or activation controls (act I, CD3/CD28 + rhIL-2 activation; act II, PHA-L+ rhIL-2 activation). Cells were analysed with a MACSQuant® Analyzer 10 and the mean fluorescence intensity was related to the vehicle control. Data are shown as mean ± SEM. *N* = 5 (F, M), *N* = 6 (A-E, G-L, N). Per biological replicate, one technical replicate was acquired. For statistical analysis of (A, M, N) one-way ANOVA with Dunnett´s correction and for (B-L) Kruskal Wallis test with Dunn´s multiple comparison test was used. Aldesleukin-treated or activated samples were compared to vehicle-treated samples. * $p < 0.05$, ** $p < 0.01$, *** $p < 0.001$ and **** $p < 0.0001$ indicate significant differences the compared samples. Abb.: CFSE, carboxyfluorescein succinimidyl ester; IL-2R, Interleukin-2 receptor; FasL, Fas ligand; TRAIL, Tumor Necrosis Factor Related Apoptosis Inducing Ligand.

Supplemental Figure 5

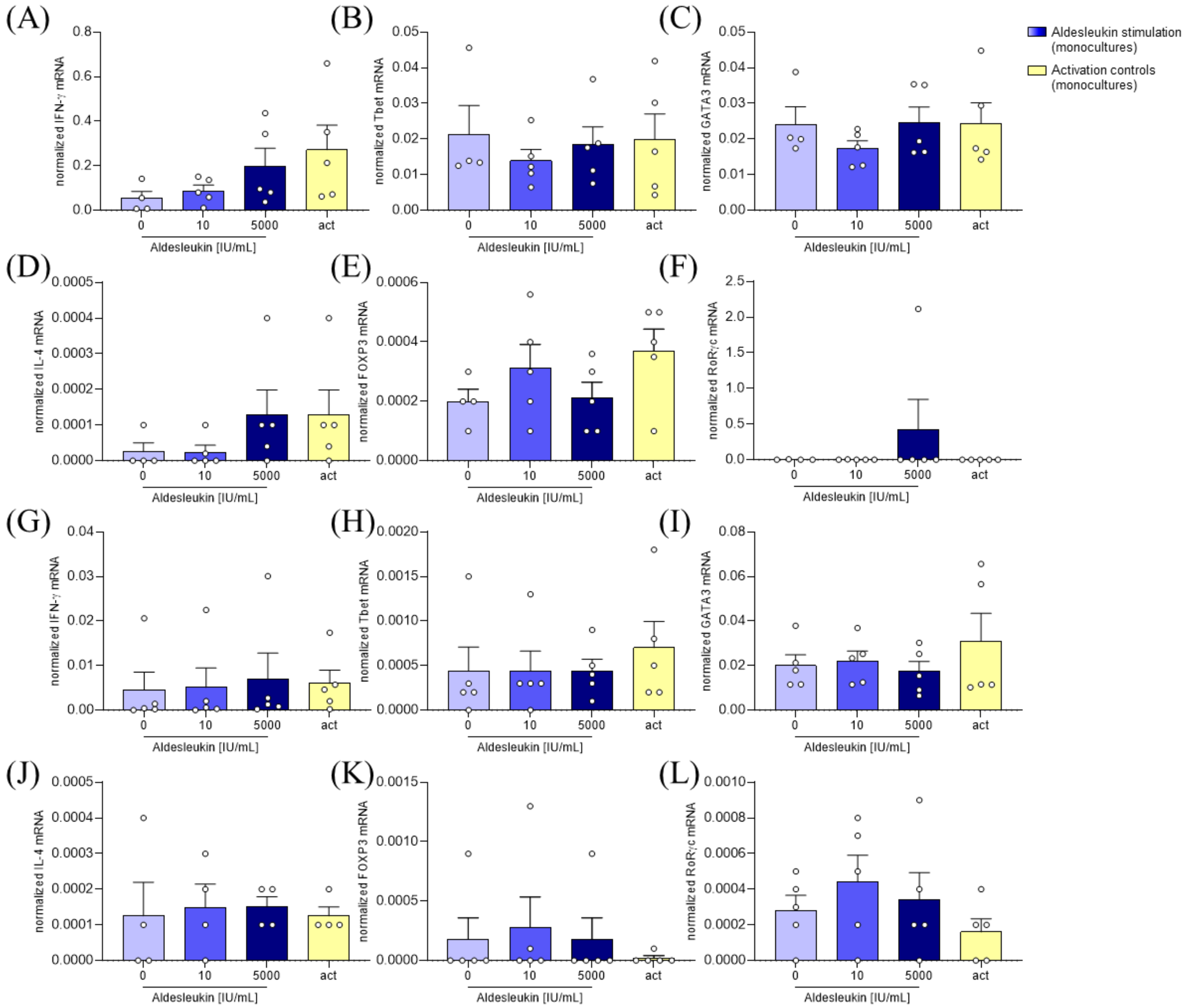

Supplemental Figure 5: Influence of aldesleukin on T helper cell polarization. T helper (Th) 1 and Th2 cells were differentiated from primary human naïve CD4$^+$ T cells. (A) Th1 cells and (B) Th2 cells were stimulated for 5 days with aldesleukin or activation control (act, PHA-L + rhIL-2). Th specific cytokines and transcription factors were determined via qPCR. For the analysis the ΔCT method was used and the expression levels were normalized to the expression of the housekeeping genes βActin and TBP-1. Data are shown as mean ± SEM. Number of biological replicates was N = 5. Two technical replicates were acquired. One-way ANOVA with Dunnett´s correction was used for statistical analyses. * $p < 0.05$, ** $p < 0.01$, *** $p < 0.001$ and **** $p < 0.0001$ indicate significant differences between Aldesleukin-treated or activated and vehicle-treated samples. Abb.: FoxP3, forkhead box P3; IFN-γ, Interferon-γ; IL-4, Interleukin-4; RORγC, RAR-related orphan receptor C; T-bet, T-box transcription factor TBX21.

Supplemental Figure 6

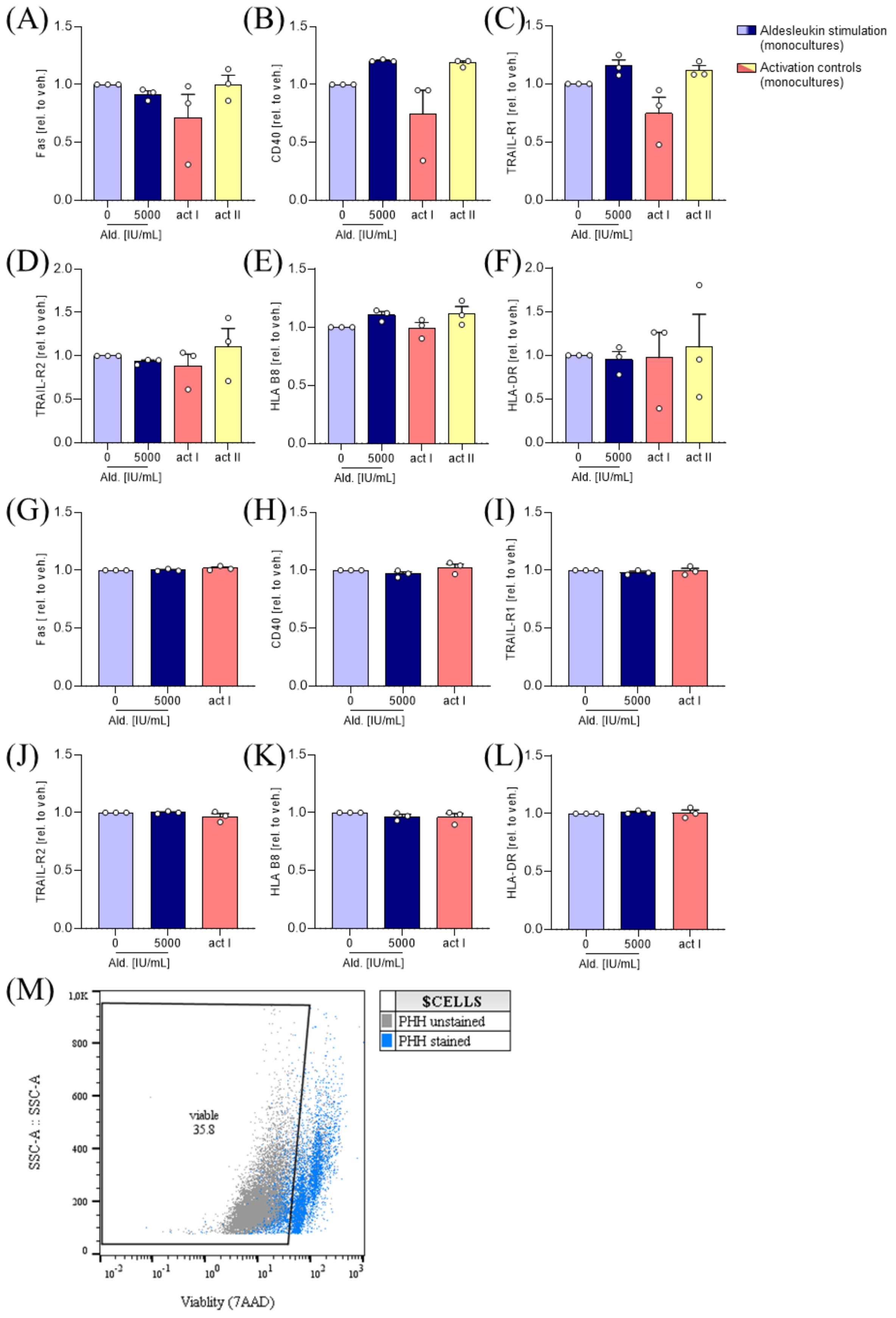

Supplemental Figure 6: Influence of aldesleukin on HepaRG and primary human hepatocyte surface marker expression. (A-F) Differentiated HepaRG cells and (G-L) primary human hepatocytes in sandwich culture were stimulated with aldesleukin or with $CD8^{+}$ T cell activation controls (act I, CD3/CD28 + rhIL-2 activation; act II, PHA-L+ rhIL-2 activation). Surface marker expression was measured via flow cytometry. $N = 3$ is the number of biological replicates and measurements were acquired with one technical replicate. Data are shown as mean ± SEM. For statistical analysis, one-way ANOVA with Dunnett´s correction was applied. $*p < 0.05$ indicates differences between the aldesleukin or activation control treated samples to the vehicle-treated samples. (M) shows a representative FACS plot for PHH viability staining. Abb.: Ald., Aldesleukin; HLA, human leukocyte antigen; TRAIL-R, TNF-related apoptosis-inducing ligand receptor.

Supplemental Figure 7

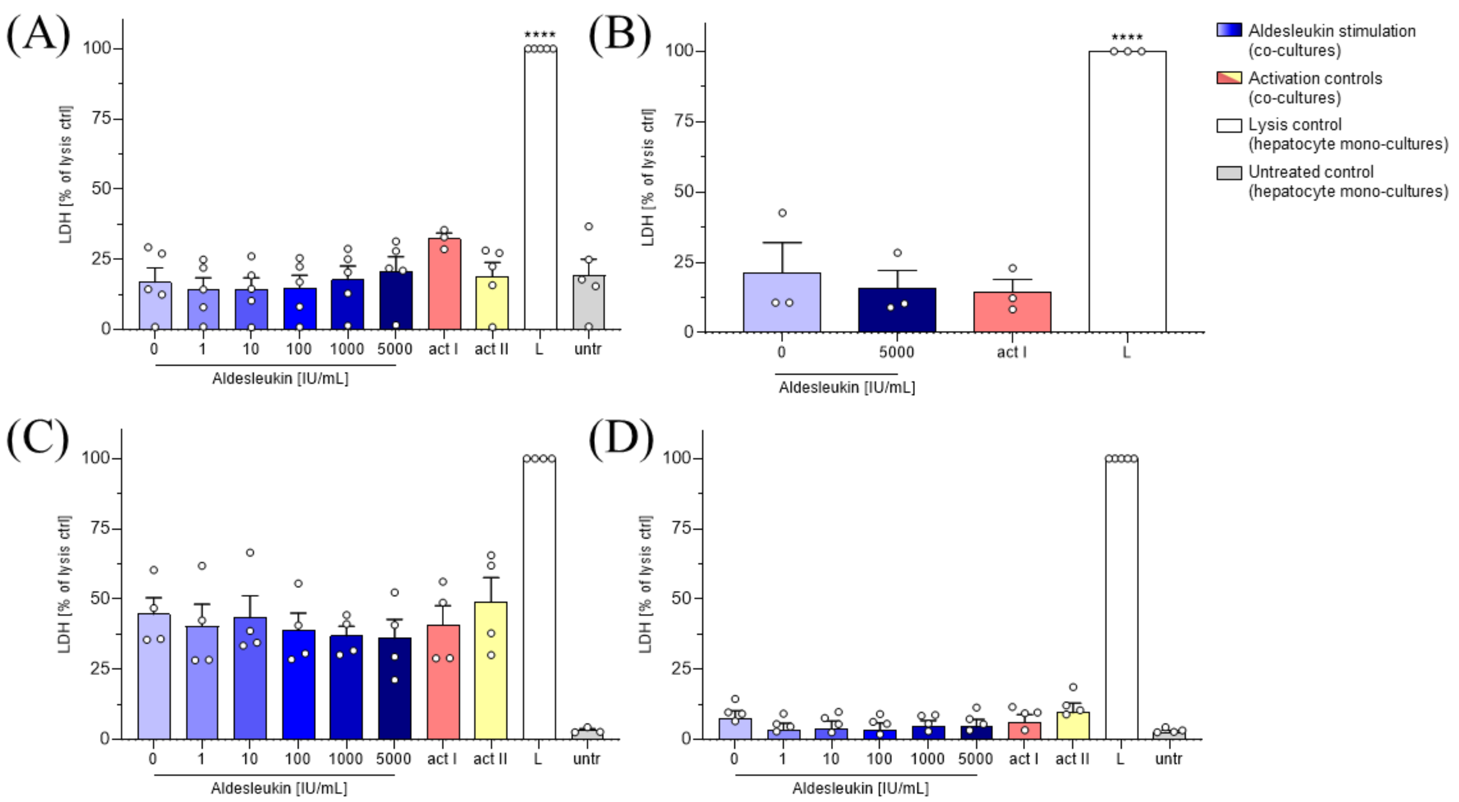

Supplemental Figure 7: Aldesleukin-mediated effects on cytotoxicity in NK/HepaRG co-cultures and hepatocyte monocultures. (A) HepaRG cells and (B) primary human hepatocytes in sandwich culture were incubated with aldesleukin or CD8^{+} T cell activation controls (act I, CD3/CD28 + rhIL-2 activation; act II, PHA-L+ rhIL-2 activation) for 5 days. (C, D) Expanded primary human NK cells were stimulated aldesleukin or activation controls (act I, CD2/CD335 + rhIL-2 activation; act II, CD2/CD335 + rhIL-15/21 activation) for 44.5 h before (C) addition onto HepaRG cells in a direct co-culture for 3.5 h or (D) NK cells were added in the upper chamber of a transwell insert and indirectly co-cultured with HepaRG cells for 2 days. Lactate dehydrogenase was determined in the supernatants and referred to the lysis control (L). Data are shown as mean ± SEM. *N* = 3 (B), *N* = 4 (C, D), *N* = 5 (A) biological replicates were acquired. In some cases, there are fewer biological replicates for the activation controls. Per biological replicate, two technical replicates were measured. For statistical analysis of (A,B,D) one-way ANOVA with Dunnett´s correction, for statistical evaluation of (C) or Kruskal Wallis test with Dunn´s multiple comparison test was used. * $p < 0.05$, ** $p < 0.01$, *** $p < 0.001$ and **** $p < 0.0001$ indicate significant differences between aldesleukin- or activation control-treated and vehicle-treated samples. Abb.: L, Triton-X-lysed HepaRG cells; LDH, lactate dehydrogenase; spont., spontaneously lysed HepaRG cells; untr., untreated HepaRG monocultures.

Supplemental Figure 8

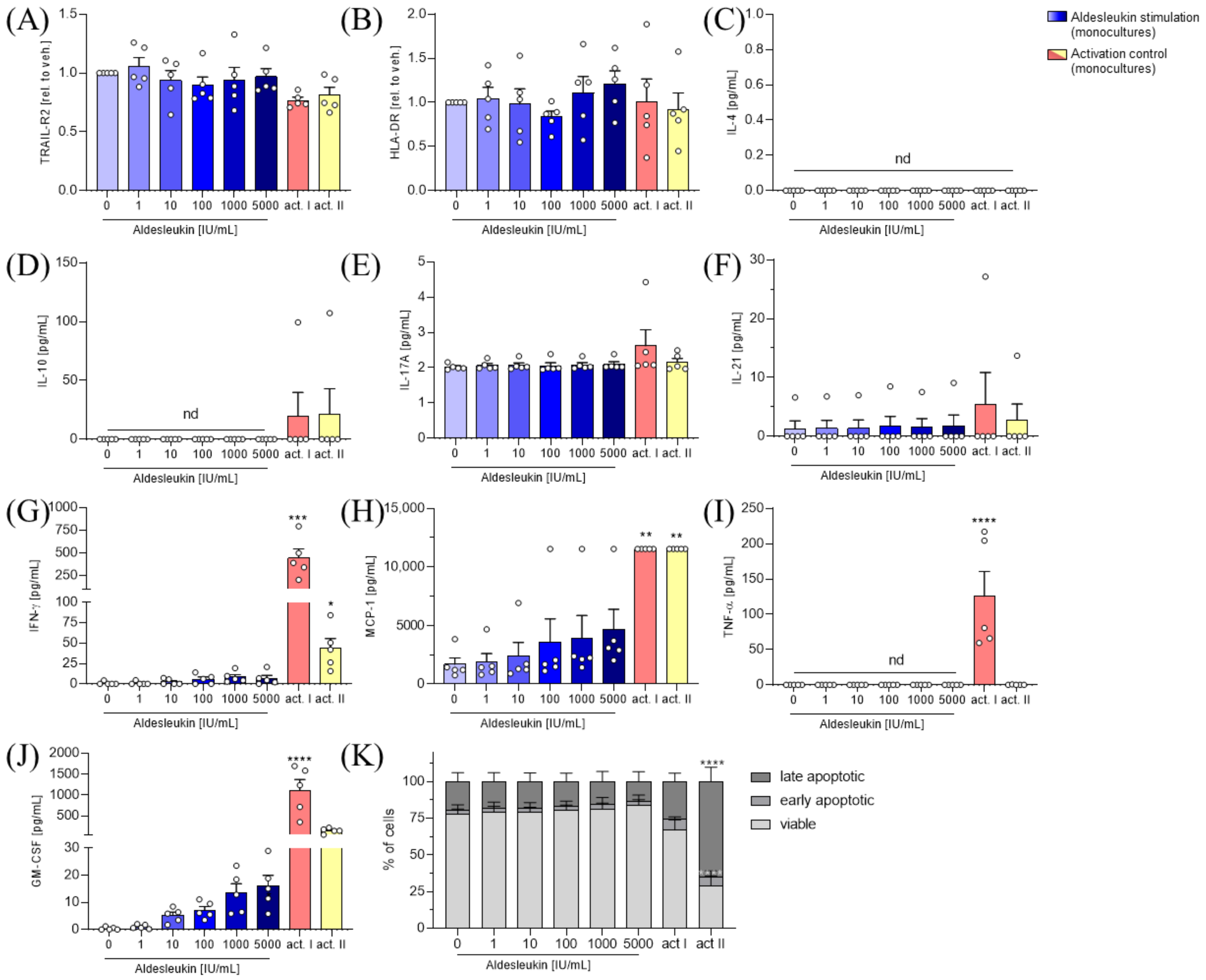

Supplemental Figure 8: Influence of aldesleukin on hepatic surface markers and molecules released to the supernatant as well as CD8⁺ T cell viability in direct CD8⁺ T cell/HepaRG co-cultures. Primary human CD8⁺ T cells from buffy coats were stimulated with aldesleukin or activation controls (act I, CD3/CD28 + rhIL-2 activation; act II, PHA-L+ rhIL-2 activation) for 3 days and co-incubated with HepaRG cells for additional 2 days. For measurement of (A, B) protein surface expression on HepaRG cells, (C-J) quantification of released proteins, and (K) viability of CD8⁺ T cells, a MACSQuant Analyzer 10 was used. Samples, in which protein concentration was below the detection limit were set to zero. Data are shown as mean ± SEM. (A-J) N = 5 and (K) N = 4 biological replicates were measured and samples were acquired as one technical replicate. For statistical analysis of (A, B, J) one-way ANOVA with Dunnett´s multiple comparison test, for (D-I) Kruskal Wallis test with Dunn´s correction, and for (K) two-way ANOVA with Dunnett´s correction was used. For (C), no statistical analysis was performed as the analyte was undetectable in all samples. * $p < 0.05$, ** $p < 0.01$, *** $p < 0.001$ and **** $p < 0.0001$ indicate significant differences between aldesleukin-treated or activated and vehicle-treated samples. Abb.: HLA, human leukocyte antigen; IFN-γ, Interferon-γ; IL, interleukin; MCP-1, monocyte chemoattractant protein 1; TNF-α, tumor necrosis factor alpha; TRAIL-R, TNF-related apoptosis-inducing ligand receptor.

Supplemental Figure 9

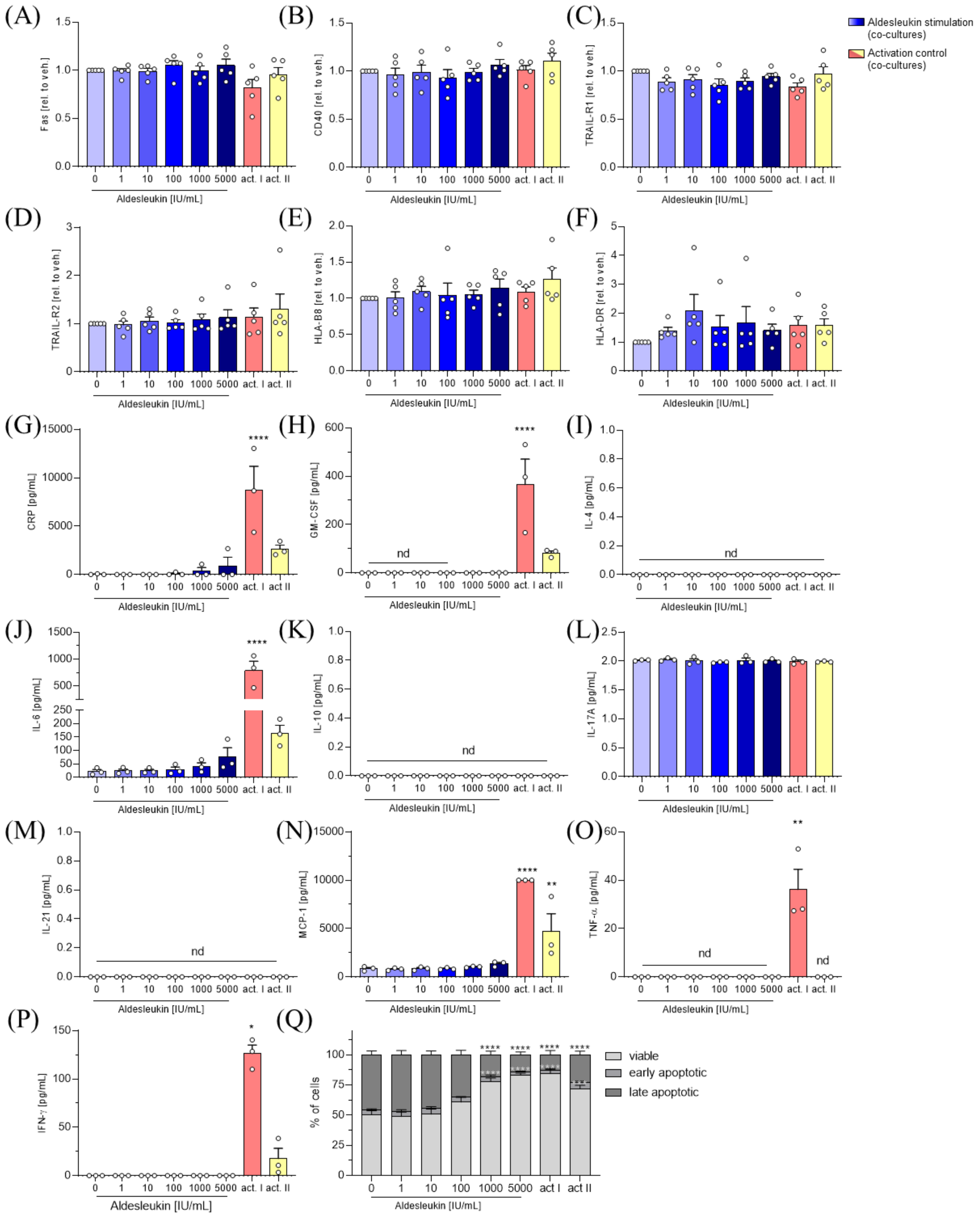

Supplemental Figure 9: Influence of aldesleukin on hepatic surface markers, molecules released to the supernatant, and CD8⁺ T viability in transwell CD8⁺ T cell/HepaRG co-cultures. Primary human CD8⁺ T cells were isolated from buffy coats and stimulated with aldesleukin or activation controls (act I, CD3/CD28 + rhIL-2 activation; act II, PHA+ rhIL-2 activation) for 2 days. Pre-stimulated CD8⁺ T cells were added in the upper chambers of transwell inserts above HepaRG cells in the lower chamber, cells were co-cultured for 3 days. For measurement of (A-F) protein surface expression on HepaRG cells (G-P) quantification of released proteins, and (Q) viability of CD8⁺ T cells, a MACSQuant Analyzer 10 flow cytometer was used. Data are shown as mean ± SEM. *N* = 3 (G-P), *N* = 4 (Q), and *N* = 5 (A-F) biological replicated were acquired. Per biological replicate, one technical replicate was acquired. For statistical analysis of (A-C, E, G, H, J, L, N) one-way ANOVA with Dunnett´s multiple comparison test, for evaluation of (D, F, O, P) Kruskal Wallis test with Dunn´s correction, and for (Q), two-way ANOVA with Dunnett´s correction was used was used. For (I, K, M), no statistical analysis was performed as the analyte was undetectable in all samples. * $p < 0.05$, ** $p < 0.01$, *** $p < 0.001$ and **** $p < 0.0001$ indicate significant differences between aldesleukin-treated or activated and vehicle-treated samples. Abb.: CRP, c-reactive protein; GM-CSF, granulocyte-macrophage colony-stimulating factor; HLA, human leukocyte antigen; IFN-γ, Interferon-γ; IL, interleukin; MCP-1, monocyte chemoattractant protein 1; TNF-α, tumor necrosis factor alpha; TRAIL-R, TNF-related apoptosis-inducing ligand receptor.

Supplemental Figure 10

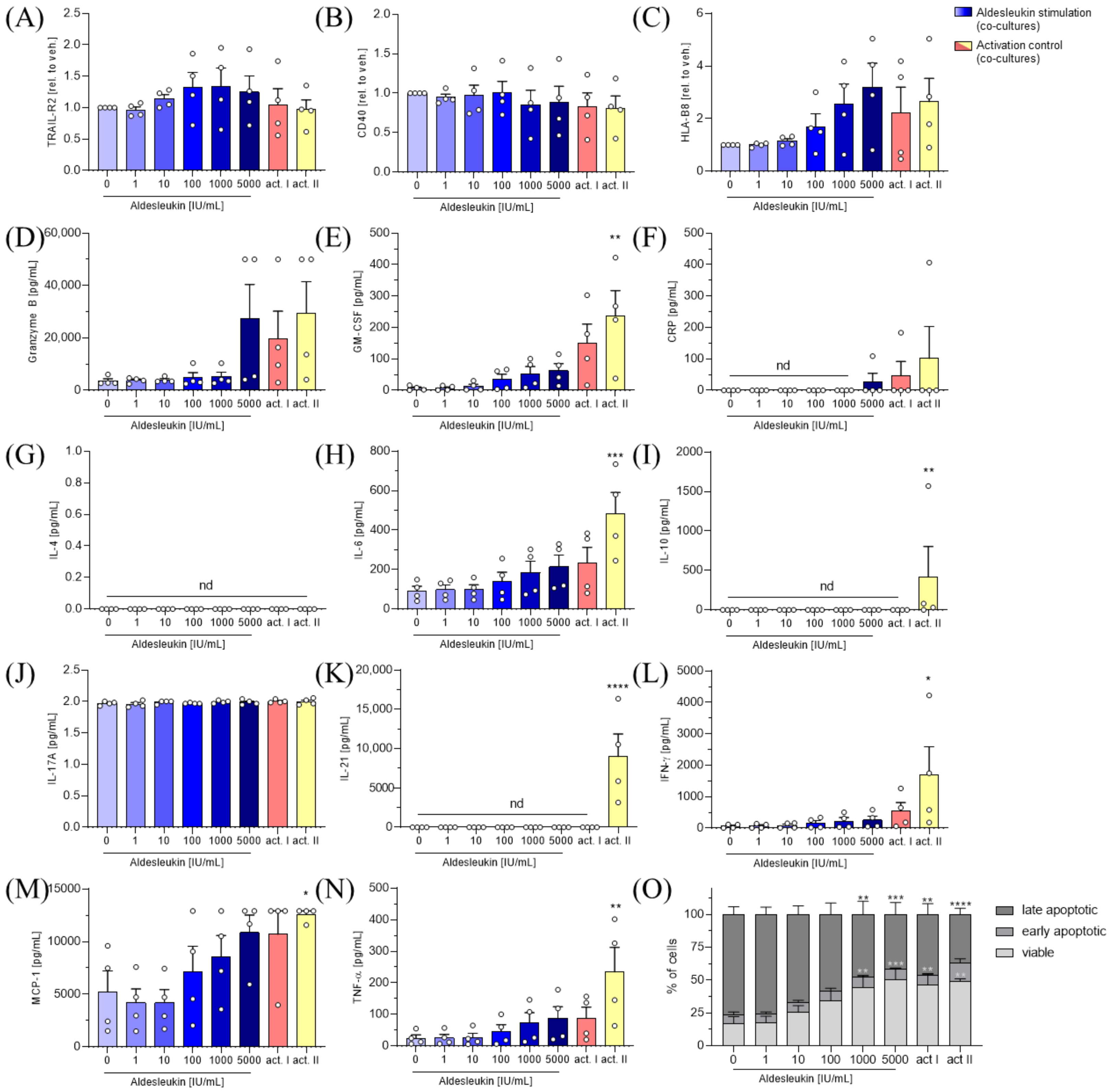

Supplemental Figure 10: Effect of aldesleukin on hepatic surface markers and molecules released to the supernatant as well as on NK cell viability in direct NK cell/HepaRG co-cultures. Expanded primary human NK cells were stimulated with aldesleukin or activation controls (act I, CD2/CD335 + rhIL-2 activation; act II, CD2/CD335 + rhIL-15/21 activation) for 44.5 h before addition onto HepaRG cells in a direct co-culture for 3.5 h. (A-C) HepaRG surface marker expression and (C-N) pro-inflammatory mediators released to the supernatant and (O) viability of NK cells, were analysed via flow cytometry, CRP was determined via ELISA. Data are shown as mean ± SEM. *N* = 4 biological replicates were measured. Samples were acquired in one technical replicate. For statistical analysis of (A-C, E, H, K, M, N) one-way ANOVA with Dunnett´s multiple comparison test, for analysis of (D, F, I, J, L) Kruskal Wallis test with Dunn´s correction, and for (O), two-way ANOVA with Dunnett´s correction was used was used was used. For (G), no statistical analysis was performed as the analyte was undetectable in all samples. * $p < 0.05$, ** $p < 0.01$, *** $p < 0.001$, and **** $p < 0.0001$ indicate significant differences between aldesleukin-treated or activated and vehicle-treated samples. Abb.: CRP, c-reactive protein; GM-CSF, granulocyte-macrophage colony-stimulating factor; HLA, human leukocyte antigen; IFN-γ, Interferon-γ; IL, interleukin; MCP-1, monocyte chemoattractant protein 1; TNF-α, tumor necrosis factor alpha; TRAIL-R, TNF-related apoptosis-inducing ligand receptor.

Supplemental Figure 11

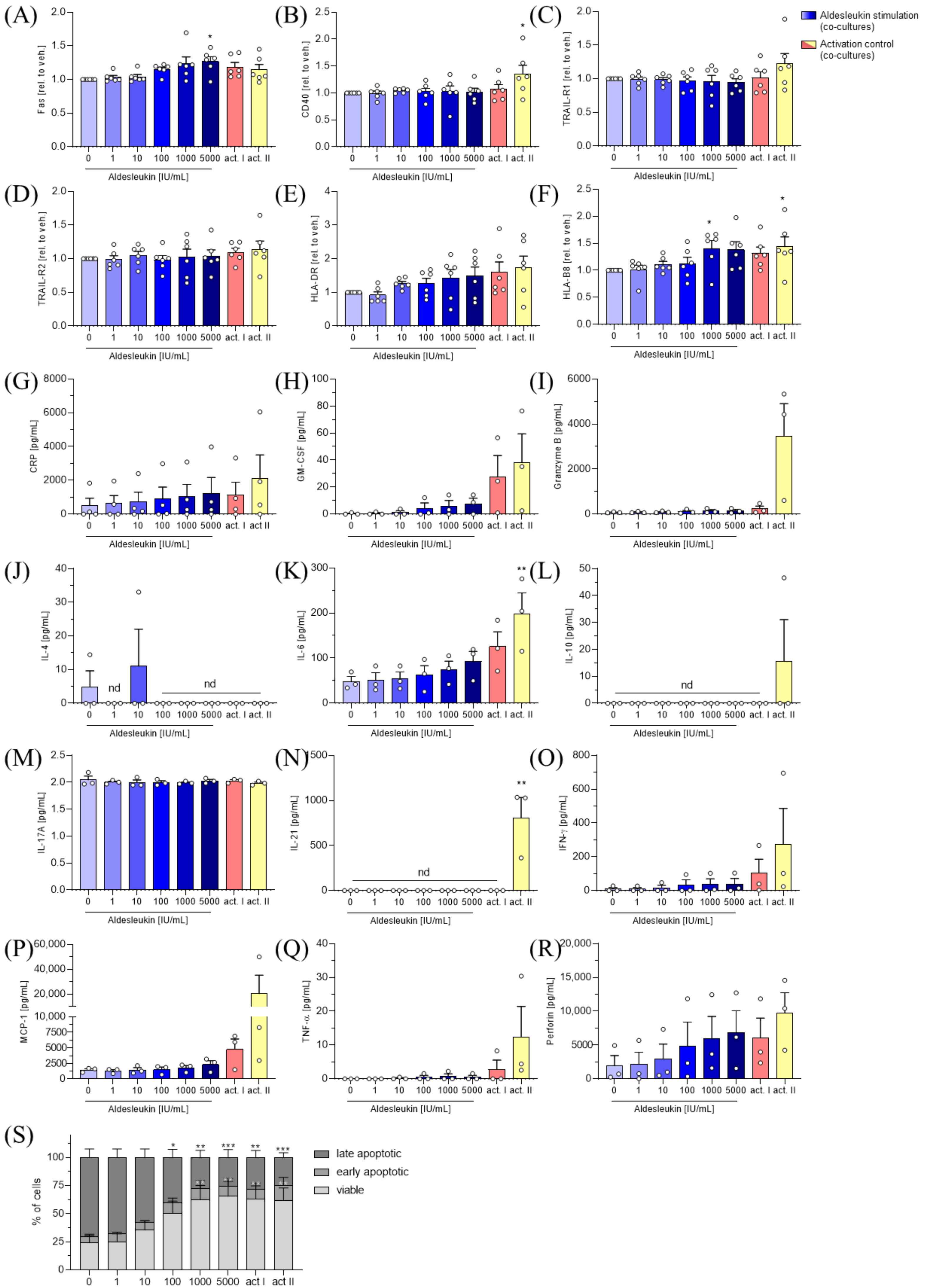

Supplemental Figure 11: Aldesleukin-mediated effects on hepatocyte surface markers, pro-inflammatory mediators, and on NK cell viability in indirect NK cell/HepaRG co-cultures. Expanded primary human NK cells were stimulated with aldesleukin or activation controls (act I, CD2/CD335 + rhIL-2 activation; act II, CD2/CD335 + rhIL-15/21 activation) and added into the upper chamber of transwell inserts with HepaRG cells in the lower chamber. Co-culture was maintained for 2 days. (A-F) HepaRG surface marker expression and (G-R) pro-inflammatory proteins released to the supernatant as well as (S) NK viability were analysed via flow cytometry, CRP was determined via ELISA. Data are shown as mean ± SEM. Number of biological replicates acquired was *N* = 3 (G-R), *N* = 4 (S) *N* = 6 (A-F). Per biological replicate, on technical replicate was measured. For statistical analysis of (B- E, H-M, Q, R) one-way ANOVA with Dunnett´s multiple comparison test, for assessment of (A, F, G, N-P) Kruskal Wallis test with Dunn´s correction, and for (S), two-way ANOVA with Dunnett´s correction was used. * $p < 0.05$, ** $p < 0.01$, *** $p < 0.001$, and **** $p < 0.0001$ indicate significant differences between aldesleukin-treated or activated and vehicle-treated samples. Abb.: CRP, c-reactive protein; GM-CSF, granulocyte-macrophage colony-stimulating factor; HLA, human leukocyte antigen; IFN-γ, Interferon-γ; IL, interleukin; MCP-1, monocyte chemoattractant protein 1; TNF-α, tumour necrosis factor alpha; TRAIL-R, TNF-related apoptosis-inducing ligand receptor.

Supplemental Figure 12

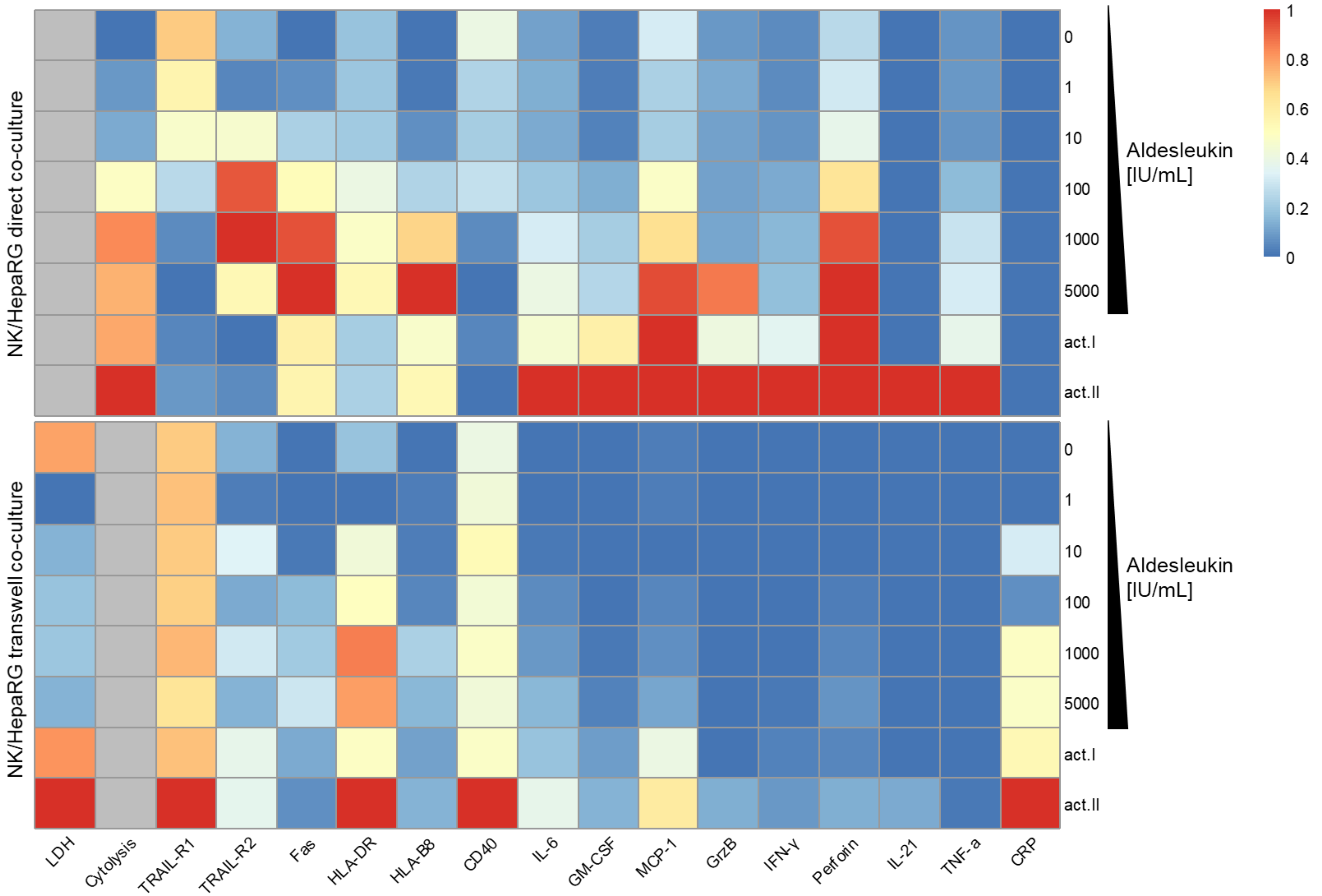

Supplemental Figure 12: Heatmap analysis of NK cell/HepaRG co-culture results. A heatmap was visualized in R representing regulation of cytotoxicity, hepatic surface marker and cytokine results direct and transwell NK/HepaRG co-culture models. For the heatmap, the median of each readout result was calculated per stimulation point and co-culture model. Data was normalized to conform to a 0 to 1 scale. For the direct co-culture, HepaRG cell cytolysis was determined as a readout for cytotoxicity, for the transwell co-culture, release of lactate dehydrogenase (LDH) was determined. Therefore, no clustering of the columns could be performed due to missing values, depicted in grey. Abb.: CRP, c-reactive protein; GM-CSF, granulocyte-macrophage colony-stimulating factor; GrZB, granzyme B; HLA, human leukocyte antigen; IFN-γ, Interferon-γ; IL-6, Interleukin-6; MCP-1, monocyte chemoattractant protein 1; TNF-α, tumour necrosis factor alpha; TRAIL-R, Tumor Necrosis Factor Related Apoptosis Inducing Ligand Receptor.

Supplemental Figure 13

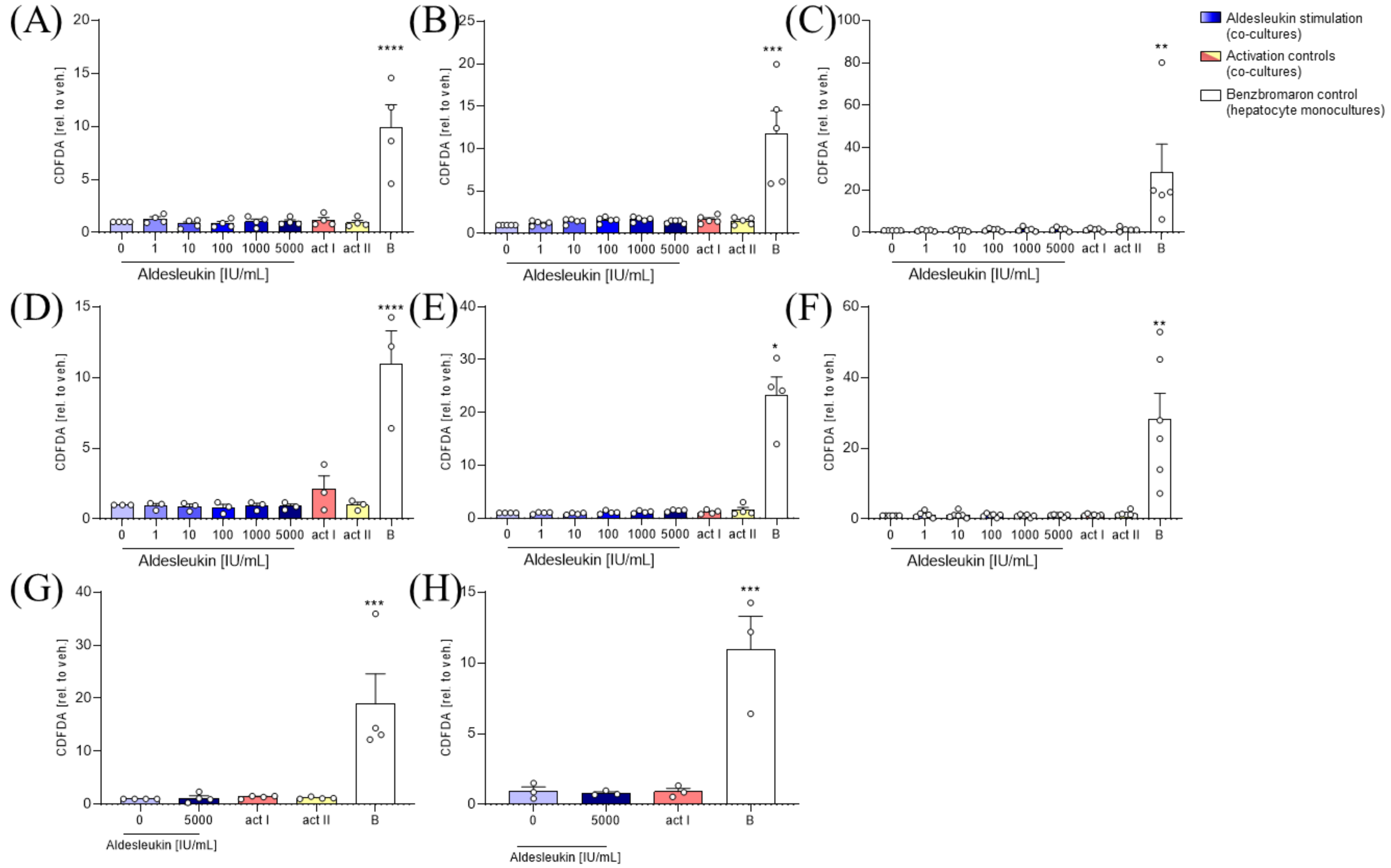

Supplemental Figure 13: Influence of aldesleukin on MRP2 function. Hyperbilirubinemia is a marker of aldesleukin-mediated hepatotoxicity. Therefore, MRP2 transporter function and bilirubin glucuronidation of hepatocytes were addressed. To determine the function of the MRP2 transporter, the retention of the fluorescent substrate 5(6)-Carboxy-2′,7′-dichlorofluorescein diacetate (CDCF, (Sigma-Aldrich, Schnellendorf, Germany) was assessed. For this, hepatpcytes were incubated at 37 °C and 5 % CO_2 in 10 mM HEPES-HBSS (Gibco™, Thermo Fisher Scientific, Oberhausen, Germany) with or without the ATPase-inhibitor benzbromarone (f.c. 100 µM, (Sigma-Aldrich, Schnellendorf, Germany). After 1 h, CDCF was added to reach a final concentration. of 5 µM and cells were incubated for additionally 90 min. Cells were then lysed in 0.1 % Triton-X (Sigma-Aldrich, Schnellendorf, Germany) in HEPES-HBSS and fluorescence intensity of the lysates was measured in black 96-well plates in the Tecan SPARK (Tecan Group, Männedorf, Switzerland) at 485/528 nm wavelength. Fluorescence values were normalized to the protein content of the lysates. For this, the Pierce™ BCA Protein Assay Kit (Thermo Fisher Scientific, Oberhausen, Germany) was performed according to the manufacturer´s protocol. (A) Primary human CD8+ T cells from buffy coats were pre-incubated with aldesleukin or activation controls (act I, CD3/CD28 + rhIL-2 activation; act II, PHA-L+ rhIL-2 activation) for 3 days and directly added onto HepaRG cells for 2 days. (B) $CD8^+$ T cells were pre-stimulated with aldesleukin or activation controls (act I, CD3/CD28 + rhIL-2 activation; act II, PHA+ rhIL-2 activation) for 2 days and added in the upper chambers of transwell inserts above HepaRG cells in the lower chamber, cells were co-cultured for 3 days. (C) Primary human CD8+ T cells were co-cultured with monocyte-derived macrophages and stimulated with aldesleukin or activation controls (act I, CD3/CD28 + rhIL-2 activation; act II, PHA-L+ rhIL-2 activation) for 3 days before addition onto HepaRG cells in direct co-cultures for 2 days. (D) Primary human $CD8^+$ T cells were pre-incubated for 3 days with aldesleukin or activation controls (act I, CD3/CD28 + rhIL-2 activation; act II, PHA+ rhIL-2 activation) before addition onto primary human hepatocytes in sandwich culture for 2 days. (E) Expanded primary human NK cells were stimulated with aldesleukin or activation controls (act I, CD2/CD335 + rhIL-2 activation; act II, CD2/CD335 + rhIL-15/21 activation) for 44.5 h before direct co-cultures with HepaRG cells for 3.5 h were performed. (F) Expanded primary human NK cells were stimulated with aldesleukin or activation controls (act. I, CD2/CD335 + rhIL-2 activation; act II, CD2/CD335 + rhIL-15/21 activation) and added into the upper chamber of transwell inserts with HepaRG cells in the lower chamber for 2 days. (G) HepaRG cell monocultures and (H) primary human hepatocyte monocultures were stimulated with aldesleukin or $CD8^+$ T cell activation controls (act I, CD3/CD28 + rhIL-2 activation; act II, PHA-L+ rhIL-2 activation) for 5 days. In all cultures, MRP2 function was determined as retention of the fluorescent surrogate CDFDA. Fluorescence intensity of cell lysates was measured in the Tecan SPARK (Tecan Group, Männedorf, Switzerland). Benzbromarone served as positive control for MRP2 transporter inhibition. Data are shown as mean ± SEM. $N = 3$ (D), $N = 4$ (A, E, G), $N = 5$ (B, C, F) biological replicates were acquired and samples were measured in one technical replicate. For statistical analysis, one-way ANOVA with Dunnett´s multiple comparison test was used. * $p < 0.05$, ** $p < 0.01$, *** $p < 0.001$ and **** $p < 0.0001$ indicate significant differences between Aldesleukin-treated or activated and vehicle-treated samples. Abb. B, Benzbromarone; CDFDA, Carboxy-DCFDA (5-(and-6)-Carboxy-2′,7′-Dichlorofluorescein Diacetate.

Supplemental Figure 14 (Legend see next slide)

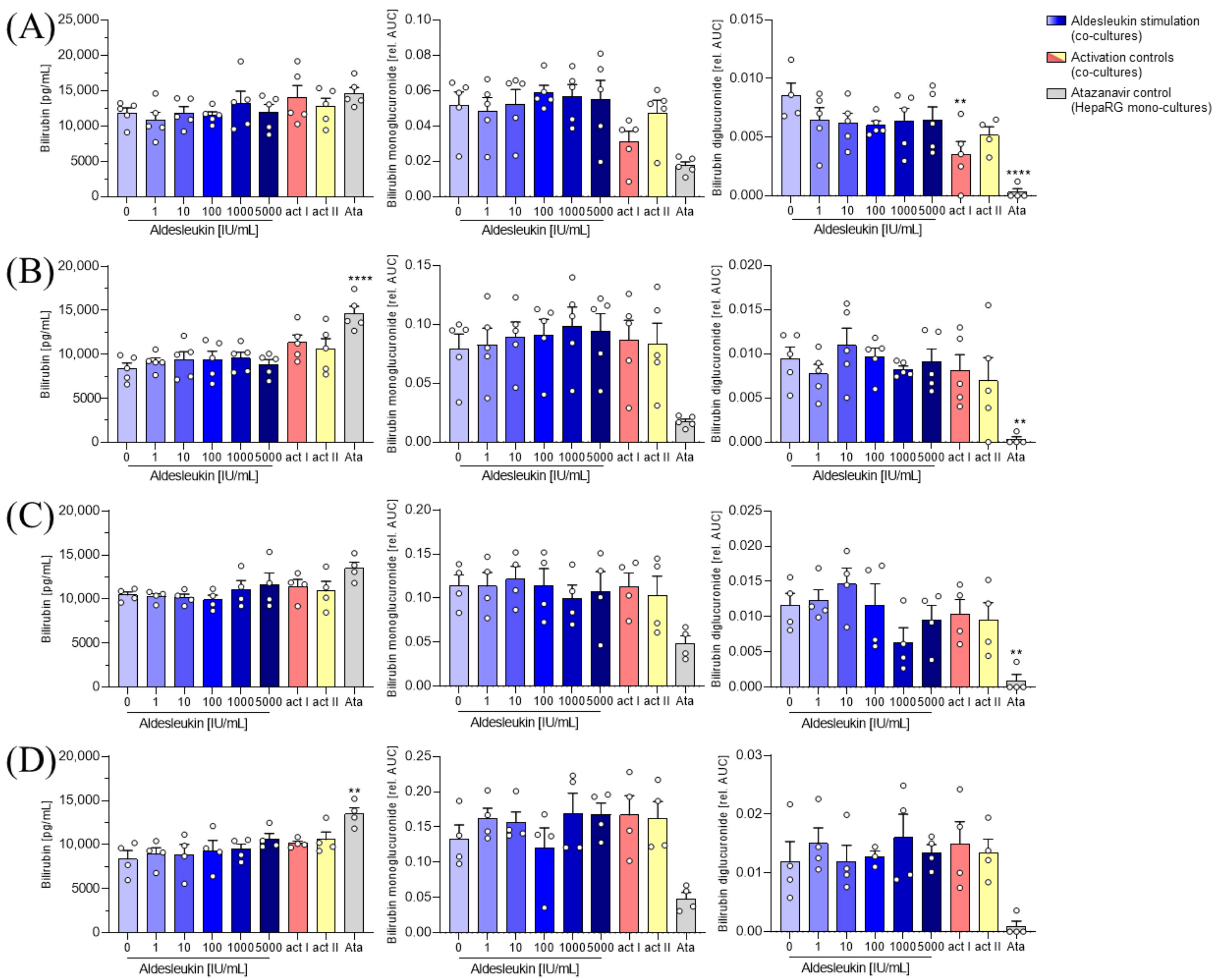

Supplemental Figure 14: Influence of aldesleukin on bilirubin glucuronidation in CD8+ T cell/HepaRG and NK cell/HepaRG co-cultures. For the measurement of bilirubin glucuronidation, a hepatocyte bilirubin conjugation assay was established. HepaRG cells from the co- and triple culture systems were washed and pre-incubated with bilirubin assay medium (HepaRG differentiation medium with 1 % DMSO and with 4 mg/mL BSA (Sigma-Aldrich, Schnellendorf, Germany) instead of FBS) supplemented with or without the UDP glucuronyl transferase inhibitor atazanavir (f.c. 2.4 µM, Sigma-Aldrich, Schnellendorf, Germany) for 1 h. Subsequently, the medium was exchanged for bilirubin assay medium supplemented with bilirubin (10 µM, Sigma-Aldrich, Schnellendorf, Germany) with or without the inhibitor atazanavir (2.4 µM, Sigma-Aldrich, Schnellendorf, Germany). After 24 h, the supernatant was collected, centrifuged and stored at -80 °C until bilirubin and bilirubin mono- and di-glucuronide detection by LC MS/MS analysis. For the analysis of bilirubin and its glucuronides, 10 µL sample were mixed with 20 µL of the IS solution (telmisartan and telmisartan-gluconoride, 50 ng/mL each in methanol), 20 µL ACN, 50 µL ACN/water (1:1 v/v) + 10 mM ammonium acetate + 1% formic acid. For calibration standards and quality control samples, 10 µL blank supernatant were spiked with 20 µL of bilirubin standard working solutions and processed like the samples. After vortexing and centrifugation, the blank supernatants were transferred to glass vials with inserts. The LC-MS/MS analysis of bilirubin and its glucuronides was carried out using an Agilent 1290 Infinity I UHPLC system (Agilent, Waldbronn, Germany) coupled to a hybrid triple quadrupole linear ion trap mass spectrometer QTRAP 6500+ (Sciex, Darmstadt, Germany) equipped with a Turbo-V-source operating in positive electrospray ionization mode. The chromatographic separation was carried out using a Kinetex C18 UHPLC column (100x2.1mm, 2.6µm, Phenomenex, Aschaffenburg, Germany), maintained at 55 °C. A gradient program was employed at a flow rate of 750 µL. Mobile phase A was water + 0.2% formic acid and mobile phase B was methanol + 0.2% formic acid. For analysis and quantification of all compounds, Analyst Software 1.7.1 and Multiquant Software 3.0.3 (both Sciex, Darmstadt, Germany) were used. The precursor-to-product ion transitions used for quantification were: m/z 585.3 → m/z 225.1 for bilirubin, m/z 761.0 → m/z 585.3 for bilirubin-glucuronide, m/z 937.0 → m/z 585.3 for bilirubin-diglucuronide, m/z 515.2 → m/z 276.2 for telmisartan and m/z 691.5 → m/z 515.2 for telmisartan-glucuronide. Calibration curves for bilirubin were constructed using linear regression with 1/x weighting. Variations in accuracy were less than 20% over the whole range of calibration, except for the lowest limit of quantification, where a variation in accuracy of 25% was accepted. Due to the lack of commercially available standards of bilirubin mono- and diglucuronide, relative quantification was performed by comparing the peak area ratios (analyte/telmisartan-glucuronide) in the samples. (A) Primary human $CD8^+$ T cells from buffy coats were pre-incubated with aldesleukin or activation controls (act I, CD3/CD28 + rhIL-2 activation; act II, PHA+ rhIL-2 activation) for 3 days and directly added onto HepaRG cells for 2 days. (B) $CD8^+$ T cells were pre-stimulated with aldesleukin or activation controls (act I, CD3/CD28 + rhIL-2 activation; act II, PHA+ rhIL-2 activation) for 2 days and added in the upper chambers of transwell inserts above HepaRG cells in the lower chamber, cells were co-cultured for 3 days. (C) Expanded primary human NK cells were stimulated with aldesleukin or activation controls (act I, CD2/CD335 + rhIL-2 activation; act II, CD2/CD335 + rhIL-15/21 activation) for 44.5 h before direct co-cultures with HepaRG cells for 3.5 h were performed. (D) Expanded primary human NK cells were stimulated with aldesleukin or activation controls (act. I, CD2/CD335 + rhIL-2 activation; act II, CD2/CD335 + rhIL-15/21 activation) and added into the upper chamber of transwell inserts with HepaRG cells in the lower chamber for 2 days. After co-culture, HepaRG cells were incubated for 24 h with 10 µM bilirubin. 2.4 µM Atazanavir served as positive control for inhibition of glucuronidation. Supernatant was analysed for bilirubin and glucuronides via LC MS/MS. Samples for which no AUC could be determined were set to zero. Data are shown as mean ± SEM. $N = 4$ (A, B) or $N = 5$ (C, D) biological replicates were acquired. For statistical analysis, one-way ANOVA with Dunnett´s multiple comparison test was used. * $p < 0.05$, ** $p < 0.01$ and **** $p < 0.0001$ indicate significant differences between aldesleukin-treated or activated and vehicle-treated samples. Abb. Ata, Atazanavir; rel. AUC, relative area under the curve.

Supplemental Figure 15

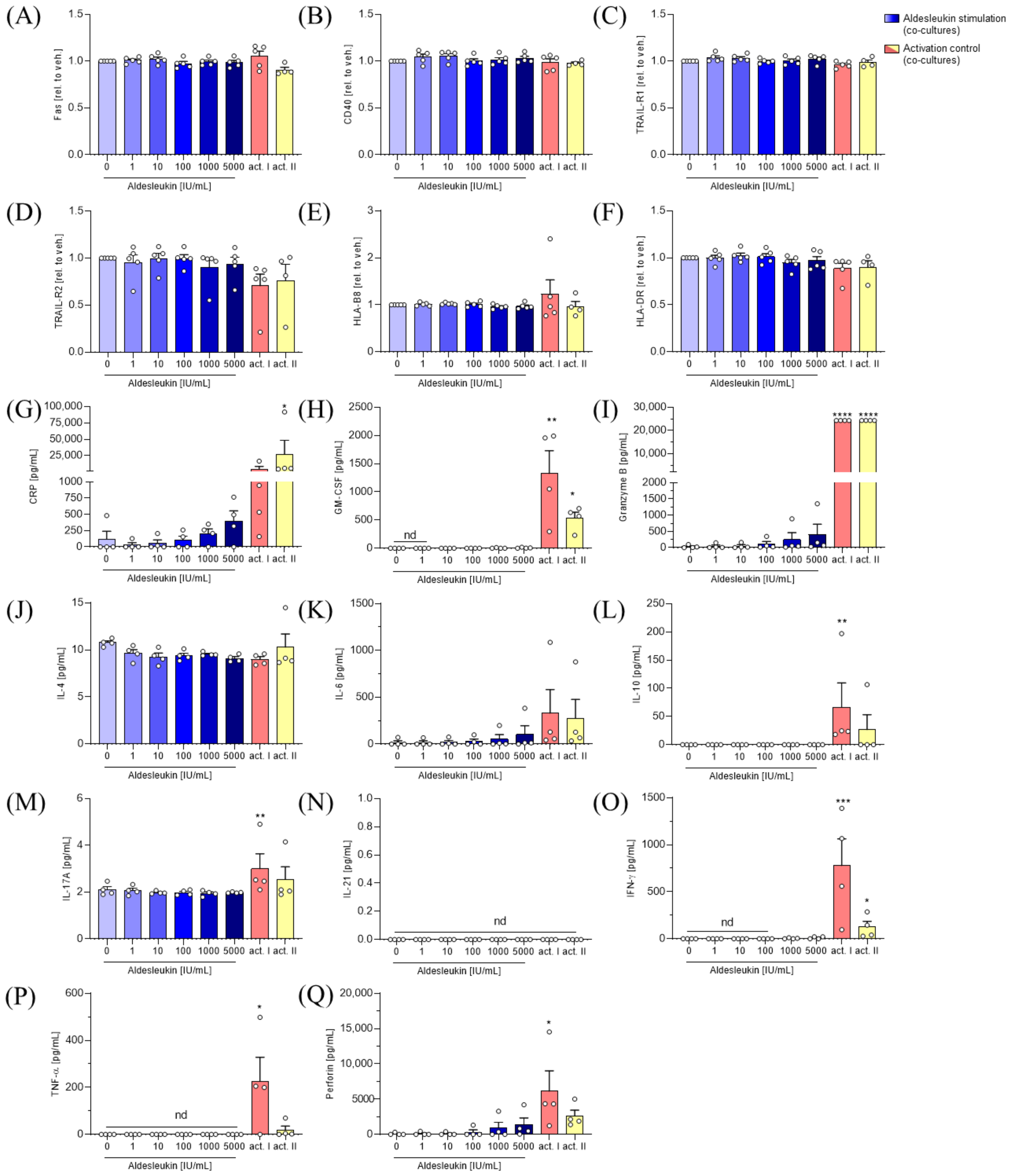

Supplemental Figure 15: Influence of aldesleukin on hepatic surface markers and molecules released to the supernatant in direct CD8⁺ T cell/primary human hepatocyte co-cultures. Primary human CD8⁺ T cells from buffy coats were treated with aldesleukin or activation controls (act I, CD3/CD28 + rhIL-2 activation; act II, PHA-L+ rhIL-2 activation) for 3 days and co-incubated with primary human hepatocytes in sandwich culture for additional 2 days. For measurement of (A-F) protein surface expression on primary hepatocytes and quantification of (G) CRP, ELISA was used and (H-Q) released pro-inflammatory proteins were measured via MACSQuant Analyzer 10. Data are shown as mean ± SEM. *N* = 4 (G-Q) and *N* = 5 (A-F) biological replicates were measured. For the surface marker analysis, act II is *N* = 4 because for one donor, there were insufficient cell numbers to conduct all the stimuli. Per biological replicate, one technical replicate was measured. For statistical analysis of (A-C, I, J, O) one-way ANOVA with Dunnett´s multiple comparison test and for evaluation of (D-H, K-M, P, Q) Kruskal Wallis test with Dunn´s correction was used. For (N), no statistical analysis was performed as the analyte was undetectable in all samples. * $p < 0.05$ and ** $p < 0.01$ indicate significant differences between aldesleukin-treated or activated and vehicle-treated samples. Abb.: CRP, c-reactive protein; GM-CSF, granulocyte-macrophage colony-stimulating factor; HLA, human leukocyte antigen; IFN-γ, Interferon-γ; IL, interleukin; MCP-1, monocyte chemoattractant protein 1; TNF-α, tumour necrosis factor alpha; TRAIL-R, TNF-related apoptosis-inducing ligand receptor.

Supplemental Figure 16

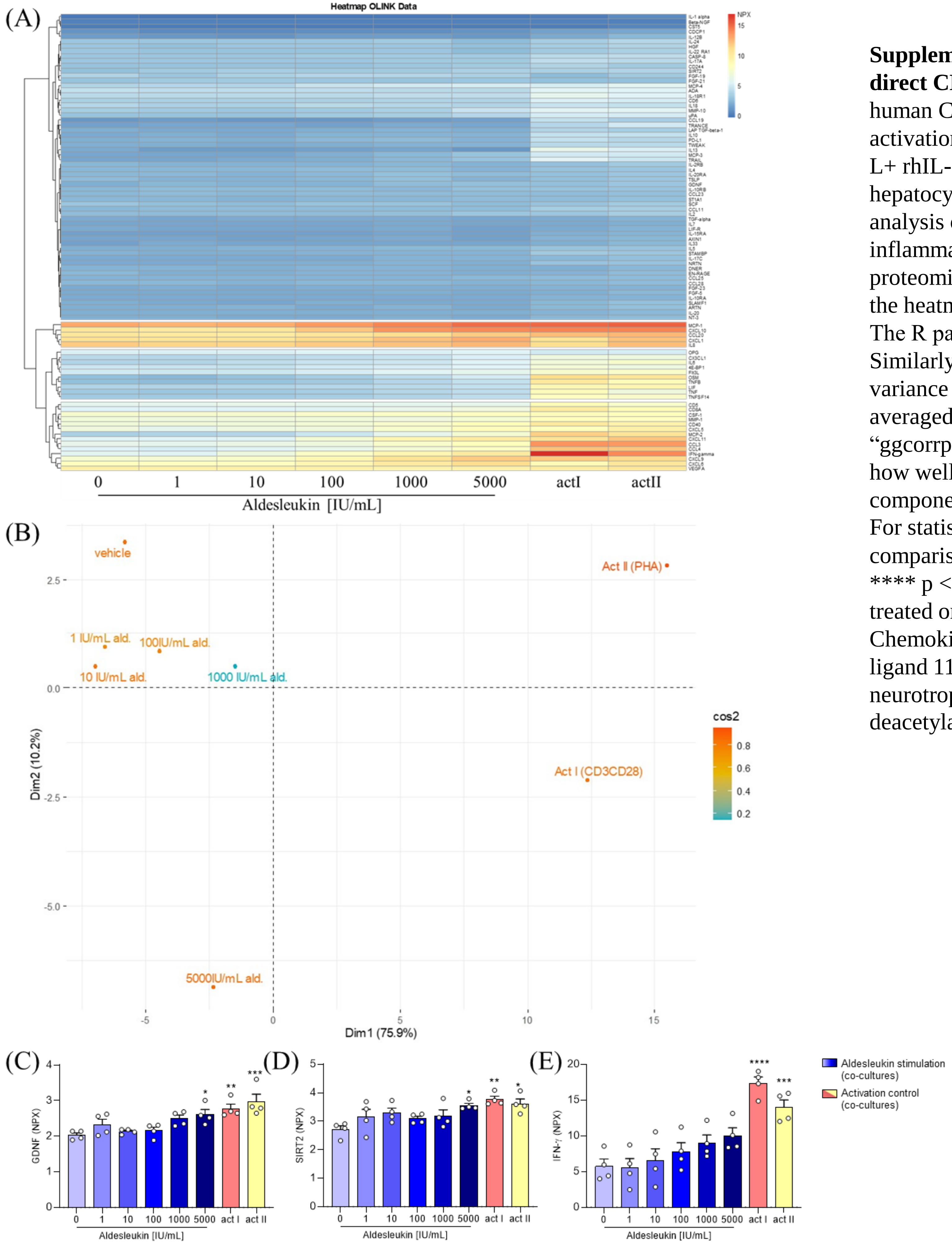

Supplemental Figure 16: Proteomic analyses of supernatants from direct CD8$^+$ T cell/primary human hepatocyte co-cultures. Primary human CD8$^+$ T cells from buffy coats were treated with aldesleukin or activation controls (act I, CD3/CD28 + rhIL-2 activation; act II, PHA-L+ rhIL-2 activation) for 3 days and co-incubated with primary human hepatocytes in sandwich culture for additional 2 days. Proteomic analysis of the supernatant was performed with the Olink® Target 96 inflammation assay (Olink, Uppsala, Sweden). (A) For the analyses of proteomic data, R version 4.2.0 (2022-04-22 ucrt) was employed. For the heatmap the averaged values of the expression data were analysed. The R packages "OlinkAnalyze" and "pheatmap" were employed. Similarly expressed proteins were clustered using the Ward's minimum variance method. (B) Principal component analysis (PCA) of the averaged values of the proteomic data. For the PCA, the packages "ggcorrplot", "factoMineR" and "factoextra" were used. Cos2 indicates how well the variables (the stimuli) are represented by the principal components. (C-E) Selected proteins from the Olink analyses. $N = 4$. For statistical analysis, one-way ANOVA with Dunnett´s multiple comparison test was applied. * $p < 0.05$, ** $p < 0.01$, *** $p < 0.001$ and **** $p < 0.0001$ indicate significant differences between aldesleukin-treated or activated and vehicle-treated samples. Abb.: CCL4, Chemokine (C-C motif) ligand 3; CXCL11, Chemokine (C-X-C motif) ligand 11; IFN-γ, Interferon-γ; GDNF, glial cell line-derived neurotrophic factor; OSM, oncostatin-M; SIRT2, NAD-dependent deacetylase Sirtuin 2.

Supplemental Figure 17

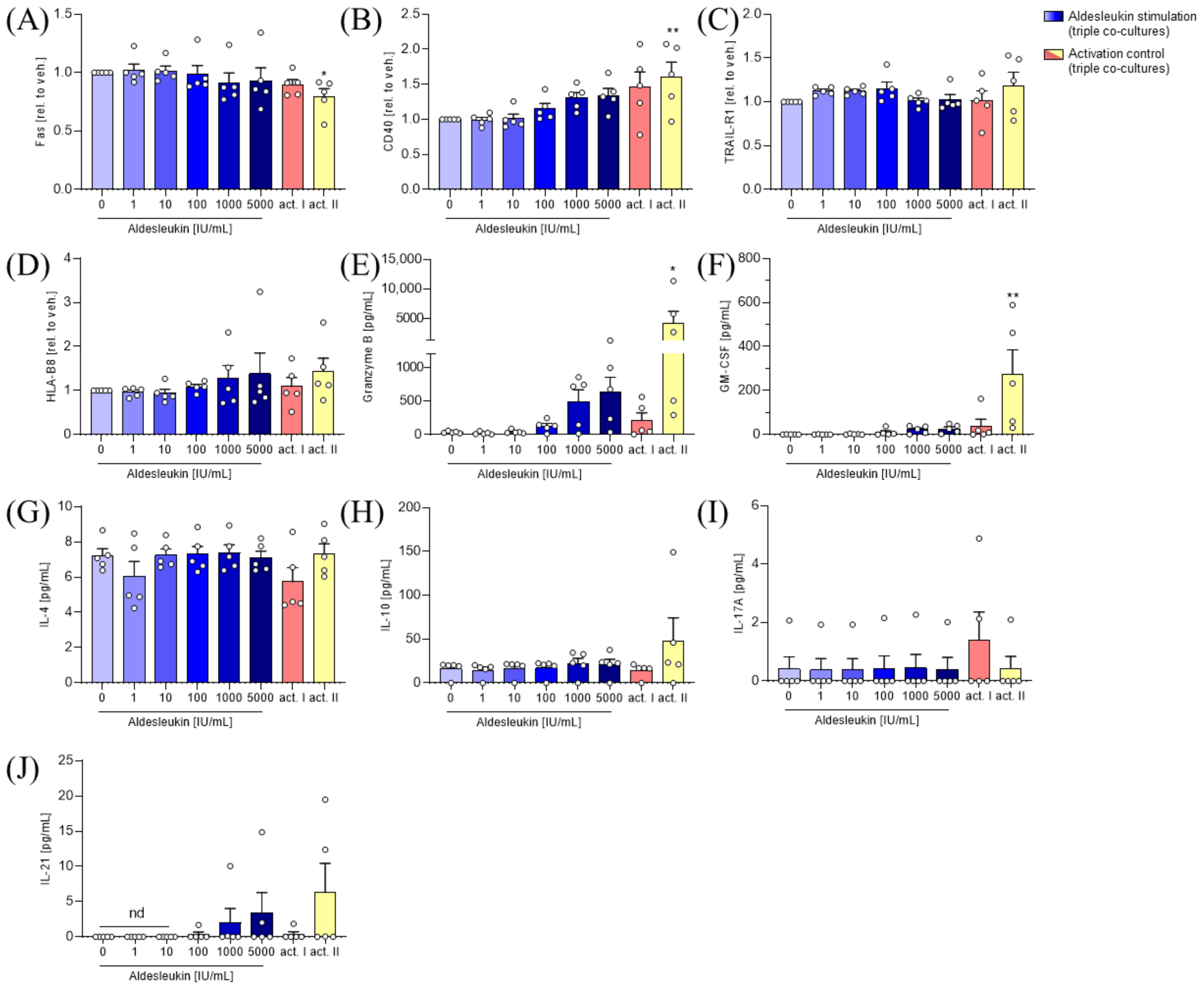

Supplemental Figure 17: Influence of aldesleukin on hepatic surface marker expression and protein release in direct co-cultures of CD8+ T cells, monocyte-derived macrophages and HepaRG cells. Primary human CD8+ T cells were co-cultured with monocyte-derived macrophages (MdMs) and stimulated with aldesleukin or activation controls (act I, CD3/CD28 + rhIL-2 activation; act II, PHA+ rhIL-2 activation) for 3 days before addition onto HepaRG cells in direct co-cultures for 2 days. (A-D) Protein surface expression on HepaRG cells and (E-K) quantification of released proteins was conducted with MACSQuant Analyzer 10. Data are shown as mean ± SEM. *N* = 5. For statistical analysis of (B, G) one-way ANOVA with Dunnett´s multiple comparison test was applied and for (A, C-F, H-J) Kruskal Wallis test with Dunn´s correction was used. * $p < 0.05$, ** $p < 0.01$ and *** $p < 0.001$ indicate significant differences between aldesleukin-treated or activated and vehicle-treated samples. Abb.: GM-CSF, granulocyte-macrophage colony-stimulating factor; HLA, human leukocyte antigen; IFN-γ, Interferon-γ; IL, interleukin; TNF-α, tumour necrosis factor alpha; TRAIL-R, TNF-related apoptosis-inducing ligand receptor.

Supplemental Table 1: qPCR primer sequences.

Name	Sequence
IL-2Rα forward	CAATGCAGCCAGTGGACCAAG
IL-2Rα reverse	TGTAGAGCCCTGTATCCCTGG
IL-2Rβ forward	CTCAGAGCATGGAGGAGACG
IL-2Rβ reverse	GTCACCTTGTCCCTCTCCAG
IL-2Rγ forward	TGGAGTGAATGGAGCCACC
IL-2Rγ reverse	CCGTTCCAGCCAGAAATACAC
TBP-1 forward	CACCACTCCACTGTATCCCTC
TBP-1 reverse	GGCACGAAGTGCAATGGTC
βActin forward	CCAACCGCGAGAAGATGA
βActin reverse	CCAGAGGCGTACAGGGATAG
GATA3 forward	GAGCAACGCAATCTGACCGAG
GATA3 reverse	GGGCGACGACTCTGCAATTC
IL-4 forward	GATTCCTGAAACGGCTCGAC
IL-4 reverse	CAACGTACTCTGGTTGGCTTC
Tbet 1 forward	AATCAGCACCAGACAGAGATG
Tbet 1 reverse	CCCGGCCACAGTAAATGACAG
FoxP3 forward	CAACATGCGACCCCCTTTCA
FoxP3 reverse	AGGCAAACATGCGTGTGAAC
RORγC forward	GCAAGACTCATCGCCAAAGC
RORγC reverse	GGTGGAGGTGCTGGAAGATC
Eomes forward	GCGCAAATAACAACAACACCCAG
Eomes reverse	CGCCATCCTCTGTAACTTCAAC

Supplemental Table 2: Surface markers and soluble proteins measured

Cell type/experiment	Markers
CD8+ T cells	CD8, CD18 (Integrin β2), CD25 (IL-2Rα), CD49a (Integrin α1), CD69, CD122 (IL-2Rβ), CD132 (IL-2Rγ), CD178 (FasL), CD253 (TRAIL)
NK cells	CD16, CD18 (Integrin β2), CD25 (IL-2Rα), CD49a (Integrin α1), CD54 (ICAM-1), CD56, CD122 (IL-2Rβ), CD132 (IL-2Rγ), CD158d (KIR2DL4), CD178 (FasL), CD192 (CCR2), CD253 (TRAIL), CD314 (NKG2D)
Th1 and Th2 cells	CD4, CD25 (IL-2Rα), CD69, CD122 (IL-2Rβ), CD132 (IL-2Rγ), CD253 (TRAIL)
Hepatocytes	CD40, CD95 (Fas), CD261 (TRAIL-R1), CD262 (TRAIL-R2), HLA-DR, HLA-B8
Proteins in the supernatant (only co-cultures)	IL-4, IL-6, IL-10, IL-17A, IL-21, GM-CSF, Granzyme B, IFN-γ, MCP-1, TNF-α, Perforin, CRP
